# Microplastics accumulate fungal pathogens in terrestrial ecosystems

**DOI:** 10.1038/s41598-021-92405-7

**Published:** 2021-07-15

**Authors:** Gerasimos Gkoutselis, Stephan Rohrbach, Janno Harjes, Martin Obst, Andreas Brachmann, Marcus A. Horn, Gerhard Rambold

**Affiliations:** 1 Department of Mycology, University of Bayreuth, Universitätsstraße 30, 95447 Bayreuth, Germany; 2 Institute of Microbiology, Leibniz University Hannover, 30419 Hannover, Germany; 3 Experimental Biogeochemistry, BayCEER, University of Bayreuth, 95448 Bayreuth, Germany; 4 Genetics, Faculty of Biology, Ludwig Maximilian University Munich, 82152 Martinsried, Germany

**Keywords:** Infection, Microbial ecology, Microbiome, Fungal ecology

## Abstract

Microplastic (MP) is a pervasive pollutant in nature that is colonised by diverse groups of microbes, including potentially pathogenic species. Fungi have been largely neglected in this context, despite their affinity for plastics and their impact as pathogens. To unravel the role of MP as a carrier of fungal pathogens in terrestrial ecosystems and the immediate human environment, epiplastic mycobiomes from municipal plastic waste from Kenya were deciphered using ITS metabarcoding as well as a comprehensive meta-analysis, and visualised via scanning electron as well as confocal laser scanning microscopy. Metagenomic and microscopic findings provided complementary evidence that the terrestrial plastisphere is a suitable ecological niche for a variety of fungal organisms, including important animal and plant pathogens, which formed the plastisphere core mycobiome. We show that MPs serve as selective artificial microhabitats that not only attract distinct fungal communities, but also accumulate certain opportunistic human pathogens, such as cryptococcal and *Phoma*-like species. Therefore, MP must be regarded a persistent reservoir and potential vector for fungal pathogens in soil environments. Given the increasing amount of plastic waste in terrestrial ecosystems worldwide, this interrelation may have severe consequences for the trans-kingdom and multi-organismal epidemiology of fungal infections on a global scale.

## Introduction

Plastic waste, an inevitable and inadvertent marker of the Anthropocene, has become a ubiquitous pollutant in nature^1^. Plastics can therefore exert negative effects on biota in both, aquatic and terrestrial ecosystems. Direct consequences of larger plastic waste for organisms range from entanglement and suffocation^2^ to intestinal obstruction^3^. Microplastic (MP) particles (< 5 mm)^4^, the most common form of fragments^5^, are taken up by various vertebrate and invertebrate species^6^, leading to extensive bioaccumulation^7^. By adsorbing a multitude of hydrophobic organic substances, plastic solid waste (PSW) may form an eco-corona and thus, inter alia, interfere with chemical communication in aquatic systems^8^ and biomagnify potentially hazardous xenobiotics in the food web^9^. Humans are constantly exposed to MP through ingestion, inhalation, and skin contact^10^. Internalised particles can, for instance, cause respiratory inflammation, lung disease and endocrinological disorders^10,11^ due to a combination of intrinsic toxicity and chemical leaching^12^.

A trending, yet poorly understood aspect of plastic pollution with potential effects on ecosystems and human health is the interaction between plastics and microbes. The hydrophobic surface of plastic waste provides an ideal environment for microbial colonisation and biofilm formation, and represents a protective ecological niche, the so-called ‘plastisphere’^13^. These epiplastic communities harbour Archaea and Bacteria^13–15^, as well as unicellular and oligocellular eukaryotes including fungi^16,17^, and have been found on plastics from marine, limnic, and fluvial ecosystems in numerous biomes from the equator to the polar regions^18^. Metagenomic studies show that MP selects for microbial communities that are different from the surrounding environment^19^, and whose composition and succession is subject to spatial^20^ and seasonal influence^19^ as well as polymer type^21^. Thus, MP represents a microhabitat with a high selectivity and plasticity, which can have effects at ecosystem level, such as the spread of antibiotic resistances through the concentration of certain lineages^22^ or changes in microbial nitrogen and carbon cycle dynamics through shifts in community structure^4^.

In this context, the role of MP as a reservoir and vector for invasive and harmful microbes is a recurring aspect in the relevant literature^11,13,23^. The durable substrates can not only massively promote adhesion, thus serving as reservoirs for pathogens, but can also be transported over long distances by wind, currents, and waves, eventually leading to the establishment of alien communities at specific destinations^24^. In marine surface waters, floating PSW acts as vector for the distribution of potentially harmful bacteria of the genus *Vibrio*^13,23^ and pathogenic serotypes of *Escherichia coli*^25^ as well as invasive algal species^24^. In addition, marine PSW has been reported to harbour microbial pathogens that can cause disease outbreaks in coral reefs^26^, fish^27^, and shellfish^18^. Although research on the plastisphere and its ecological impact has made enormous progress in recent years, we are far from an integral understanding. Reasons for the limited state of knowledge on the plastisphere holobiome are, on the one hand, the focus on prokaryotic communities, and, on the other hand, the somewhat limited consideration of terrestrial ecosystems.

Fungi are the ideal group of organisms for studying microbial plastic colonisation in terrestrial systems, as they are particularly well adapted to life in the plastisphere due to their adsorptive nutrition mode, apical growth^28^, invasive growth forms^29^, biofilm formation, and the secretion of hydrophobic proteins (hydrophobins)^30^. Most phylogenetically higher (non-zoosporic) fungi are not bound to the aqueous phase for their propagation, produce far more biomass in certain soils than prokaryotes^28,31^, and in principle can therefore systematically colonise soil-deposited plastic waste. Fungi are early colonisers of drifting MP^20^, part of polymicrobial biofilms e.g., on domestic plastic surfaces^32^, and have also been isolated from landfill plastics^31^. Pathogenic fungal taxa such as *Candida*, *Fusarium* and *Rhodotorula* are known to occur on plastic surfaces of medical^33^, industrial^11^ and household appliances^32^. Thus, MP potentially can play a role in the accumulation and spread of fungal pathogens in soil environments receiving massive influx of PSW, such as home gardens, roadsides, agricultural soils, and landfills^34^. So far, studies on such effects of MP have been carried out in aquatic systems and remote areas without considering the immediate human environment.

We addressed these knowledge gaps by providing first in-depth insights into fungal communities of the plastisphere biome and by evaluating the role of MP as a carrier of potentially pathogenic fungi in terrestrial ecosystems. Our three hypotheses were that soil-deposited MP (1) is readily colonised by fungal biofilms, (2) hosts a distinct mycobiome different from that of the surrounding bulk soil, and (3) accumulates a variety of pathogenic species, including opportunistic human pathogens. We conceptualised an operational design allowing the comparative study of soil-inhabiting and plastic-associated assemblages in situ in human settings. Therefore, we collected five biological replicates from the topsoil of five different sites with high human activity and high-level plastic pollution within the municipal boundary of Siaya, Western Kenya. The sites included two landfills, a marketplace, a roadside, and a courtyard. ITS metabarcoding was applied to decipher the fungal community diversity of the plastic and soil (sub)samples obtained through selective subsampling, while scanning electron microscopy (SEM) and confocal laser scanning microscopy (CLSM) were used to visualise patterns of fungal plastic colonisation. Finally, we used trait data from multiple sources (meta-analysis) to construct a functional profile of the dominant fungal phylotypes of the plastisphere. From these findings, we demonstrate the role of MP as a selective microhabitat and ‘hot spot’ for potentially human pathogenic fungal species in terrestrial ecosystems in general, and the immediate human environment in sub-Saharan Africa in particular.

## Results

### Fungal colonisation of MP

**Figure 1:**
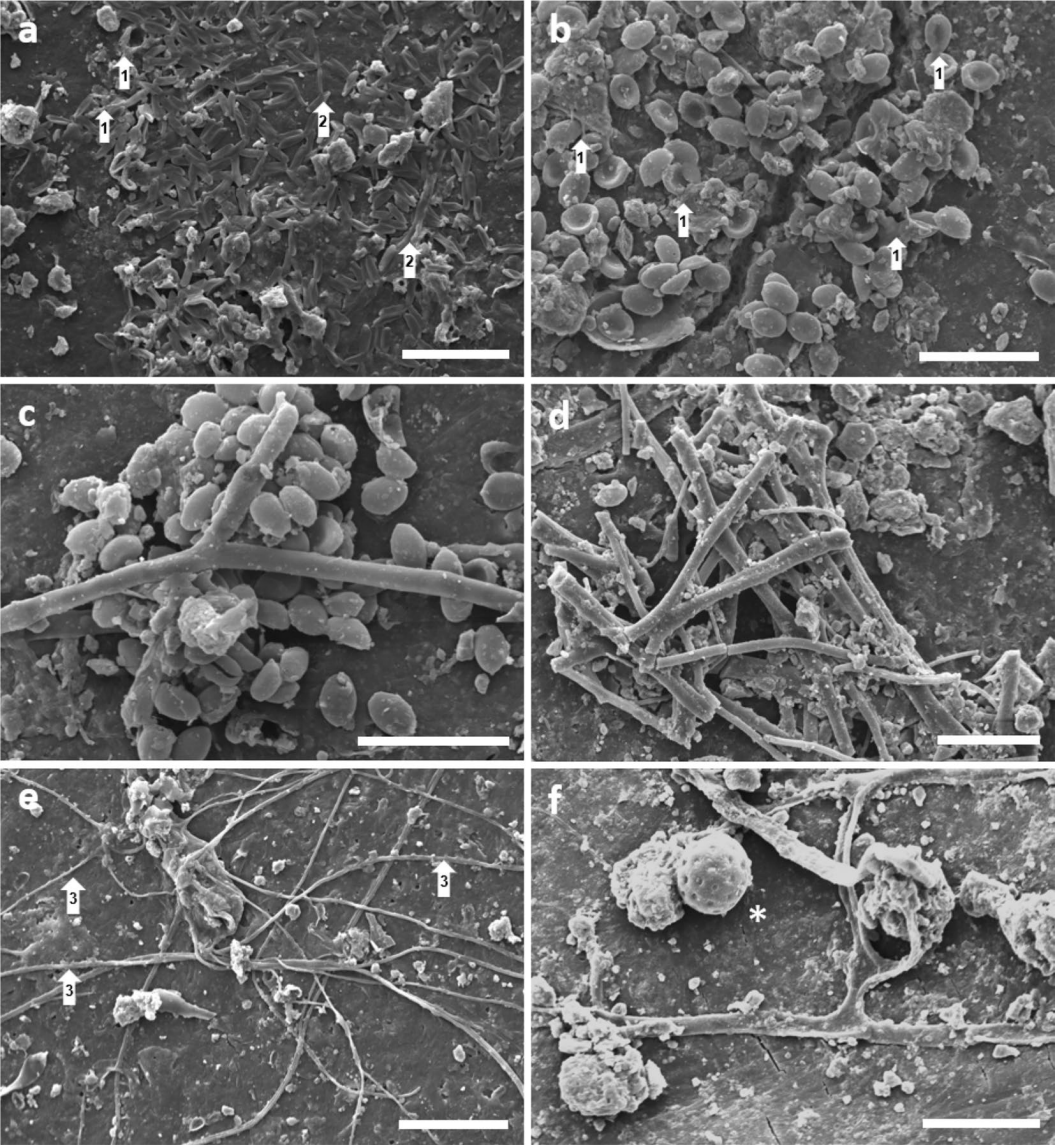
Fungal colonisation of MP fragments visualised by SEM. White arrows indicate specific structures. (**a**) Mat of fungal conidia, including potentially germinating spores (1), closely attached to the plastic surface presumably through a form of self-produced mucilage (2). (**b**) Numerous conidia lining a crack in the plastic surface. (**c**) Clumping of conidia in association with a hypha. (**d**) Mycelial meshwork. (**e**) Extensive intertwined hyphal filaments adhering to the plastic surface via small peripheral bulges (3). (**f**) Conidia-producing hypha (conidiophore) with exposed vesicle after discharge (asterisk). Scale bars are 30 µm.

To visualise fungal growth and biofilm formation on plastic debris, MP fragments were subjected to SEM and CLSM analysis. SEM micrographs proved strong colonization of plastic surfaces by diverse fungal organisms for all analysed samples (Fig. 1a–f). The plastisphere contained fungal propagules and organismal structures of filamentous fungi, including vegetative and reproductive hyphae, while yeasts or yeast-like cells were not detected. Asexual fungal spores (conidia) of different morphologies were encountered in large clusters and mats (clumping) (Fig. 1a–c), presumably attached to the polymer matrix through a secreted mucilage (Fig. 1a,b). Various morphotypes of hyphae were either observed as loose fragments scattered across the surface or forming extensive filamentous networks (Fig. 1e) and compact mycelia (Fig. 1d). Apparently, hyphae adhered to the plastic surface through small peripheral cell wall protrusions (Fig. 1e). The presence of putatively germinating spores (Fig. 1a) and a conidiogeneous hypha (conidiophore) after spore liberation (Fig. 1e) indicated fungal reproduction and propagation within the plastisphere. CLSM images evidenced nucleic acids covering the entire MP particle surface and fungal extracellular polymeric matrix (ECM; e.g., polysaccharides, glycoproteins) distributed as localised and congregated patches across the plastisphere (Supplementary Fig. 7). Overall, all stages of fungal biofilm formation were observed, including propagule adsorption, attachment, initial formation of microcolonies, ECM production, establishment of hyphal networks, spore maturation, and release.

### Effect of substrate type and location

**Figure 2:**
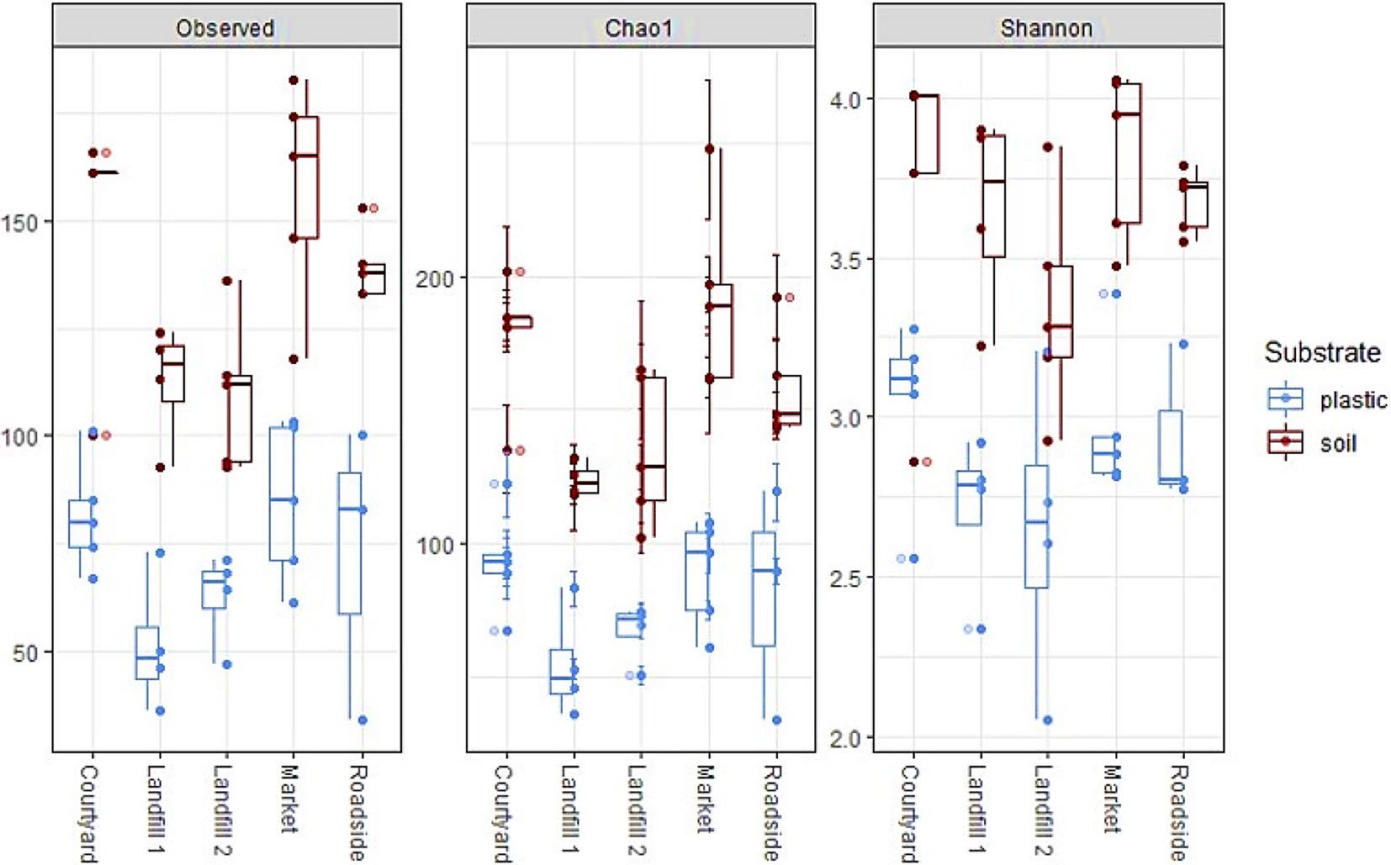
Alpha diversity metrics for the entire rarefied dataset according to substrates and sites. Samples were rarefied at the smallest library size (2496 sequences). Significance testing (ANOVA) revealed significantly higher fungal richness (observed, Chao1; both p < 0.01) and Shannon diversity (p < 0.01) of soil in comparison to plastic samples (Supplementary Fig. 2).

**Figure 3:**
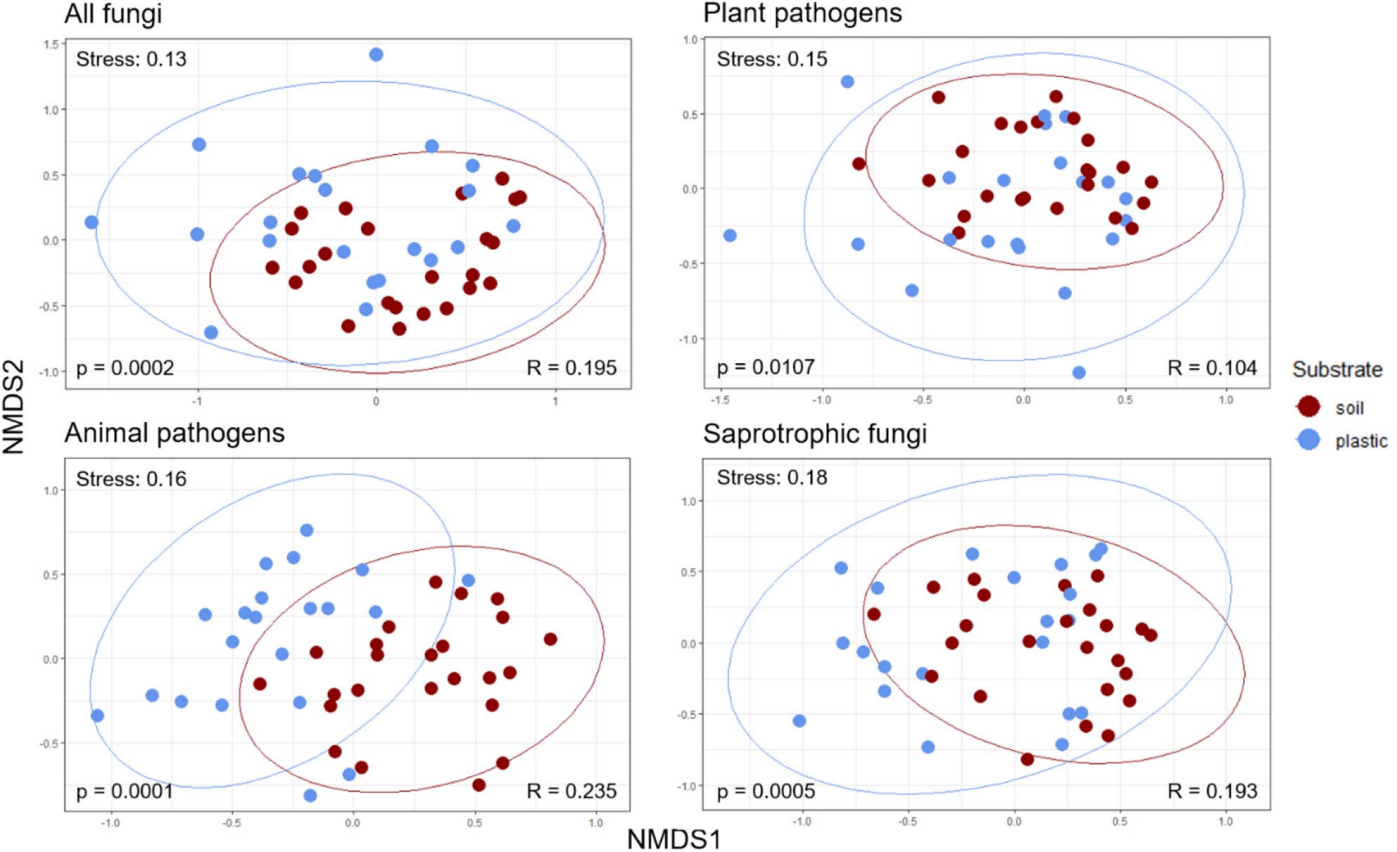
Variation in fungal community composition between plastic and soil visualised by NMDS. All four plots are based on Bray–Curtis dissimilarity matrices of square root transformed relative abundances of OTUs. Taxa subsets of the most dominant ecological guilds were compiled following meta-analysis and included only OTUs classified to genus or species level. Ellipsoids represent a 95% confidence interval surrounding the data points of each factor group. Significant variation between substrates was tested by PERMANOVA (included p-values) (Supplementary Table 4), while similarities within and between groups were additionally assessed using ANOSIM (sample statistic ‘R’) (Supplementary Table 6). NMDS ordination stress values included.

After quality control and filtering, a total of 1,444,874 high-quality ITS sequences were obtained with an average of 32,128 reads per sample. These sequences were clustered to 566 operational taxonomic units (OTUs) from which 563 were assigned to taxa within the kingdom Fungi. The three unassigned OTUs were excluded from downstream analyses. All recovered taxa were present in the soil samples, while the plastic samples contained 76% of fungal taxa detected in soil, indicating that the plastisphere mycobiome represents a subset of the soil fungal community. Fungal species richness (ANOVA p < 0.01), Shannon diversity (p < 0.01) and (Pielou’s) evenness (p < 0.01) were significantly higher in the soil than in the plastic samples across all five sites (Fig. 2; Supplementary Table 1), pointing toward a microcosm effect of MP. Beta diversity (using Bray–Curtis dissimilarity on square-root transformed read counts) of epiplastic and soil fungal communities was assessed for all recovered fungi, and taxa subsets of the three most dominant ecological guilds in terms of OTU richness, namely plant pathogens (101 OTUs), saprotrophs (86) and animal pathogens (75). Substrate type had a significant effect on fungal community structure of all fungi (PERMANOVA, p < 0.01), plant pathogens (p < 0.05), saprotrophs (p < 0.01) and animal pathogens (p < 0.01) (Fig. 3; Supplementary Table 4), demonstrating that epiplastic and soil fungal communities were distinct. Sample ordination was visualised by non-metrical multidimensional scaling (NMDS) (Fig. 3). The factor ‘site’ significantly differentiated most epiplastic communities from one another (pairwise PERMANOVA), except for two groups (Supplementary Table 6). Factor groups were mostly homogeneously dispersed (PERMDISP, Supplementary Table 6), allowing for a clear interpretation of the results (location effects).

### The plastisphere mycobiome

Between 34 and 127 distinct fungal OTUs could be identified within a single plastic sample. Ascomycota dominated the plastisphere with 84% of fungal reads recovered from plastic assigned to this phylum, followed by Basidiomycota, unassigned fungi, Chytridiomycota and Glomeromycota with 6%, 6%, 4% and < 1%, respectively. At class level, most reads were assigned to Dothideomycetes (62%) followed by Sordariomycetes (18%) and unassigned fungi (6%). All other classes accounted for < 5% of reads each. Functional meta-analysis conducted exclusively with epiplastic fungi classified to genus or species level (58% of OTUs) revealed the presence of nine ecological guilds and six trophic modes, respectively. Across all sites and plastic samples, biotrophic-antagonistic was by far the most abundant trophic mode (Ø 92%), followed by saprotrophic (Ø 7%) and extremely low proportions of fungi adopting a commensalistic (neutral) or mutualistic lifestyle. Plant and animal pathogens were predominant across all sites and samples with an average of 51% and 40% respectively. Saprotrophs were the third most abundant classified ecological guild (Ø 7%), while the proportion of parasites of lichens and fungi was negligible.

**Figure 4:**
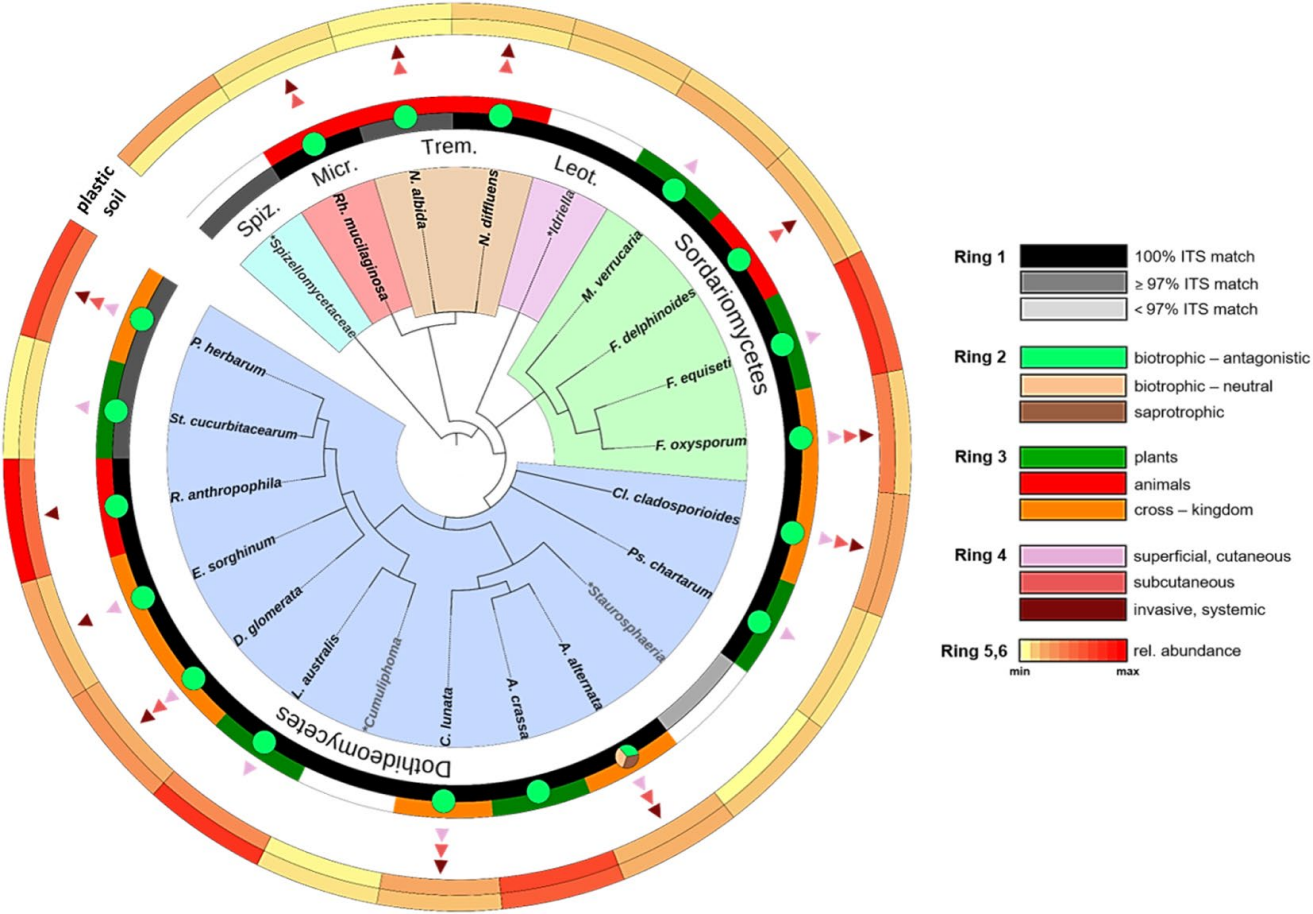
Phylogenetic distributions within the PCM. Displayed are those 22 OTUs accounting for ≥ 0.5% of plastisphere reads and contributing ≥ 0.3% to the plastic/soil dissimilarity based on SIMPER analysis with occurrence on MP in every site. OTUs classified at the species level (black font) were annotated with trait data. OTUs classified to at genus or higher level were not annotated (grey font). (*) indicates ‘unclassified’. Branch colours code for the indicated fungal classes, where Spiz. = Spizellomycetes, Micr. = Microbotryomycetes, Trem. = Tremellomycetes, Leot. = Leotiomycetes. Ring 1 indicates the success of OTU assignment based on the presence of a representative type strain or isolate ITS sequence match in the UNITE database. Ring 2 shows the most likely trophic mode for each taxon. Ring 3 displays the host (kingdom) range of the fungal taxa. The presence of at least one triangle in Ring 4 indicates potential human pathogenicity of the respective fungus, while the number and colour of the triangles code for possible virulence based on the known infection sites of the human body. Ring 5 and 6 indicate the relative abundance of each taxon on soil and plastic, respectively.

To detect and characterise the most dominant fungal phylotypes of the entire plastisphere, we selected those fungal OTUs that were most abundant (≥ 0.5% of plastisphere reads), discriminant (≥ 0.3% contribution to the dissimilarity between plastic and soil based on SIMPER analysis) and frequent (occurrence in samples of all sites). We found a plastisphere core mycobiome (PCM) consisting of 22 omnipresent phylotypes that synergistically contributed to approx. 22% of the dissimilarity between plastic and soil mycobiomes. This PCM accounted for 65% of the reads on plastic, despite comprising only 5% of epiplastic OTUs. Overall, 17 out of the 22 dominant phylotypes had 100% sequence similarity with sequences deposited in the UNITE database. PCM taxa showed a close evolutionary distance, as 21 of 22 fungi belonged to Dikarya (Ascomycota and Basidiomycota). Again, Ascomycota and Dothideomycetes were the most representative phylum and class, respectively. While all ascomycetes were filamentous fungi (Pezizomycotina), the three basidiomycetes were yeasts. All Dothideomycetes were dematiaceous (melanised fungi), while also the three basidiomycetous yeasts were so-called black yeasts, indicating that the trait of melanisation was prevalent in the plastisphere. Finally, 18 out of 22 PCM taxa could be classified to species level with a high overall classification success, and characterised with respect to their trophic mode, host range and potential human pathogenicity (virulence) (Fig. 4, Supplementary Table 9).

### Accumulation of fungal pathogens

All species of the PCM can adopt a pathogenic lifestyle as they were classified as biotrophic-antagonistic on plants, animals, or both (Fig. 4, Supplementary Table 9). Every single species within the PCM (except *A. crassa*) includes strains that were reported as opportunistic human pathogens that can infect different sites of the human body, including skin and/or other superficial areas (superficial, cutaneous), subcutaneous tissue (subcutaneous) and deep tissue and/or multiple organs (invasive, systemic) (Fig. 4, Supplementary Table 9). The most dominant fungal species on MP, accounting for more than 13% of reads, was *Remotididymella anthropophila*, a *Phoma*-like fungus recently discovered from a clinical sample of the human respiratory tract, followed by the phytopathogenic *Leptosphaerulina australis* (7%) and the multi-host infecting *Phoma herbarum* (> 6%) (Fig. 4, Supplementary Table 9). Among the most dominant plastisphere fungi were also the ubiquitous, soil-borne cross-kingdom pathogens *Fusarium oxysporum*, *Alternaria alternata*, and *Didymella glomerata* (Fig. 4), all of which are also known to cause mild or severe infectious diseases in humans, including fusarioses, eumycetoma, and phaeohyphomycoses (Supplementary Table 9). However, these taxa showed similar or even higher relative abundances in the soil mycobiome. Widespread air-borne moulds such as *Cladosporium cladosporioides* and *Curvularia lunata* were also part of the PCM (Fig. 4). These fungi occasionally act as allergens but can also cause more severe bronchopulmonary infections and cutaneous and subcutaneous lesions (Supplementary Table 9). Finally, we found the cryptococcal yeasts *Naganishia albida* and *N. diffluens*, and the red-pigmented yeast *Rhodotorula mucilaginosa* to be dominant on MP, all of which appear as important animal pathogens and can cause serious pathologies in humans (Supplementary Table 9).

**Figure 5:**
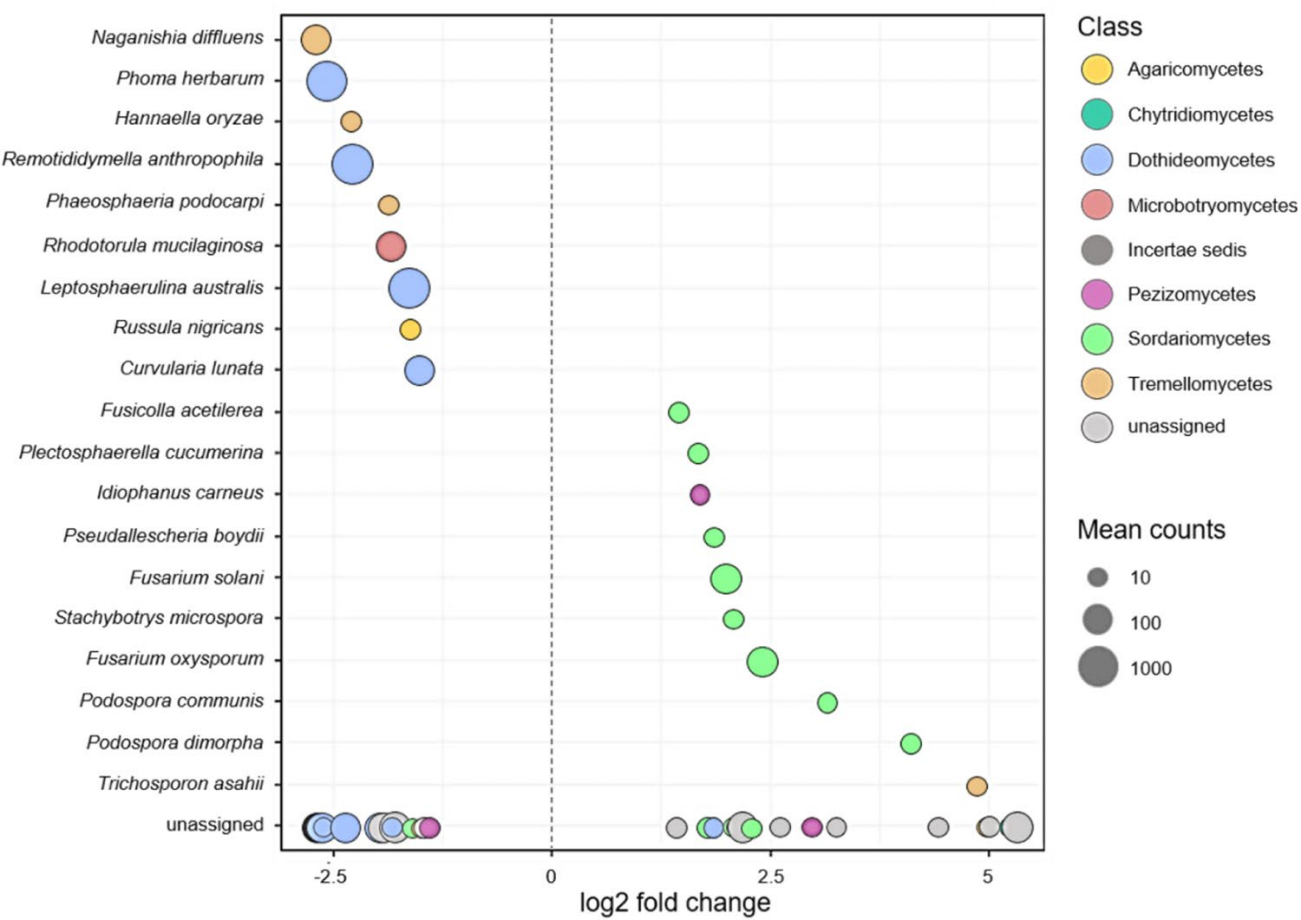
Differentially abundant fungal species of the soil and plastisphere mycobiome identified by DESeq2 analysis. Negative values indicate a significantly (p < 0.05) higher abundance of species on MP. Species are plotted according to their log2 fold change differential abundance and color-coded according to classes. Size factor normalisation was implemented in DESeq2. Bubble size corresponds to the mean read counts across all samples.

We performed a differential abundance analysis (considering only OTUs with a relative abundance of ≥ 0.1% across all samples) to detect phylotypes characterising the plastisphere and thus check for pathogens that are what we refer to as “plastiphilic”. DESeq2 test confirmed the enrichment of the above-described most abundant opportunistic human pathogens *P. herbarum*, *R. anthropophila*, *C. lunata*, *N. diffluens* and *Rh. mucilaginosa*, the rather rare plant pathogens *L. australis*, *Hannaella oryzae*, *Phaeosphaeria podocarpi* and the non-pathogenic *Russula nigricans* as well as several other unassigned species through MP (Fig. 5). Overall, the plastisphere mycobiome is thus primarily characterised by melanised pathogens of the classes Dothideomycetes and Tremellomycetes known to be able to cause cutaneous, subcutaneous, and systemic diseases in humans.

## Discussion

Unravelling microbial life on plastic waste is essential for predicting and assessing its potential ecological effects on nature and humans. The study of plastisphere organisms helps to understand the role of MP as a microhabitat and vector of potentially pathogenic microbes. Evidence for the presence of pathogenic bacteria on plastics already exists for aquatic biomes^23,25^ and is currently in the focus of microplastics research. Fungi have been largely neglected in this endeavour despite their enormous diversity^35^, ecological significance^36^, affinity for plastic^16,17^ and impact as pathogens^37,38^. Aggravatingly, terrestrial ecosystems in general and human settings in particular have been extremely poorly covered, despite being the first and major sinks of MP^39,40^. We addressed both knowledge gaps by disentangling plastisphere mycobiomes from plastic-polluted soils at various locations within the municipal boundary of Siaya, Western Kenya. Importantly, we focused particularly on ‘hot spots’ of plastic pollution that are characterised by high human utilisation, including open dumpsites, a roadside, and a marketplace.

Our detailed, database-driven documentation of the study design and measurement data of the sample processing workflow, starting with sample collection, allows a detailed quality assessment of each object with respect to FAIR++ guiding principles^41,42^. High amounts and purity of the recovered nucleic acids from both substrate types were indicative of the applicability of the extraction kit used. Applied ITS metabarcoding entails constraints regarding the coverage of rare taxa^43^, while the used primer pair displays inherent preference for specific fungal taxa^44,45^, possibly resulting in their over-amplification, and hence over-estimation of their abundance. By applying a filter criterion whereby all OTUs with less than 100 reads or an occurrence in only one sample were removed from the dataset, we mitigated stochastic effects and increased the probability of obtaining truly plastic-associated fungi, while placing the focus on abundant taxa that are likely to be of ecological relevance. Eliminating rare OTUs further mitigated the effects of ITS primer bias, poor clustering, and potential cross-contamination, increasing the informative value of the data. Saturated species rarefaction and accumulation curves (Supplementary Fig. 4) indicated adequate representation of the most abundant members and sufficient sampling depth, a prerequisite for accurate exploration of highly diverse mycobiomes^45^. Therefore, despite the underestimation of rare taxa, it can be argued that the scope of sampling and employed methodology were adequate for exploring the mycobiomes of the target substrate types.

Phenotypic and genotypic data provided complementary proof for fungal colonisation of soil-deposited MP, unveiling a rich fungal plastisphere. Accordingly, we accept our first hypothesis. Given the high fungal richness in this study (430 OTUs) as well as the disclosed spectrum of biofilm and ontogenetic stages (Fig. 1), the terrestrial plastisphere must be regarded a suitable ecological niche for a diversity of fungi. Despite the examination of a significantly lower amount of plastic, the observed richness in this study is considerably higher than reported by Lacerda et al.^17^ (135 OTUs) and Kettner et al.^16^ (347) for floating plastic in marine surface waters, implying that MP may attract greater fungal diversity in the soil environment than in aquatic ecosystems. Diverging factors such as sampling design, polymer type, ambient mycobiota or contaminant dynamics, however, impede comparability between plastisphere studies. As MPs harboured only a subset of 430 (76%) OTUs, bulk soil apparently represents the source biome of plastic colonisers with a total of 563 OTUs (100%). Significantly higher species richness, diversity, and evenness of the soil mycobiome implied a microcosm effect of MP (Fig. 2, Supplementary Fig. 6). Presumably, plastic selects fungi with certain adhesive abilities and enriches them, while at the same time less adapted (generalist) phylotypes are excluded, resulting in a compact, uneven plastisphere mycobiome, as already described for bacteria on marine plastic litter^14^. Beta diversity analyses evidenced complete differentiation of fungal communities and the prevalent ecological guilds between MP and soil (Fig. 3). Concordantly, other studies have also found differences in the microbiome structure of plastispheres and their surrounding natural environment^16,19^. Consequently, and consistent with our second hypothesis, MPs in soil systems represent selective, artificial microhabitats that assemble distinct fungal communities and pathospheres. These microcosmic consortia may allow new possibilities of transitivity, niche sharing and interaction on the allochthonous substrates.

Site characteristics apparently affected fungal plastic colonisation as the composition of plastisphere communities also varied with the sampling location (Supplementary Fig. 8). A PERMDISP analysis indicated differences in community structure as a function of locality (location effect), except for two cases that showed higher variability in composition (dispersion effect) (Supplementary Table 6). Site dependence of plastic-associated microbial communities has already been reported in other studies^15,19,20^. Despite the rather site-specific composition of epiplastic communities, certain phylotypes occurred across all sites (Fig. 4). This dichotomy suggests the relevance of local soil properties and ambient species pools to plastisphere structure as well as the existence of omnipresent systematic colonisers. Whether these potent colonisers are part of the regional mycobiota or are spread in these systems by invading MP remains to be resolved.

The selection of the most common, abundant and discriminant plastisphere fungi revealed a compact core mycobiome consisting of a few Dikarya, primarily Ascomycota, that accounted for the vast majority of reads and showed systematic incidence on MP across all sites (Fig. 4). Similarly, the finding of a core microbiome has been reported from plastisphere studies of bacteria and eukaryotes in marine waters^14,20^. A proximal explanation for the predominance of Ascomycota within the PCM is their similarly high relative number in the bulk soil that likely hosts the origin community of plastic colonisers. However, Kettner et al.^16^, Oberbeckmann et al.^19^ and De Tender et al.^20^ also reported that Ascomycota is the dominant fungal phylum on plastic samples from marine waters, which may indicate mechanistic factors such as a higher dispersal ability, a broad niche spectrum and a high adaptability of these fungi^36^.

Our meta-analysis revealed that the core taxa on MP belong to plant, animal, and multi-host pathogens. Remarkably, most dominant fungal species across all plastispheres are opportunistic human pathogens that are responsible for a wide range of human mycoses (Fig. 4, Supplementary Table 9). The classification as a potential human pathogen was based exclusively on the phylogenetic assignment to DNA reference marker sequences that have been published in clinical and scientific publications. Opportunistic virulence is context-dependent and thus not generalisable from experimental setups. Moreover, virulence traits of opportunistic fungi, such as melanisation, are exaptations (‘dual-use’ traits) that exist as such in other non-pathogenic species and lineages^46^. Due to the symplesiomorphic nature of these traits, pathogenicity tests are unnecessary to identify opportunists.

Among the most dominant plastisphere fungi, we detected pathogenic species that to our knowledge have never been found on plastics, like the filamentous, phytopathogenic *Myrothecium verrucaria*, *Fusarium equiseti* (Sordariomycetes) and *Alternaria crassa*, *Pseudopithomyces chartarum* and *Stagonoropsis cucurbitacearum* (Dothideomycetes) as well as the zoopathogenic mould *Fusarium delphinoides* and the yeast *Naganishia albida* (Fig. 4). Importantly, we also found potentially human pathogenic fungi that have been previously linked to plastic colonisation in other contexts: the trans-kingdom pathogens *Fusarium oxysporum* and *Alternaria alternata* form biofilms on indoor^47^ and landfill plastics^48^, members of *Cladosporium*, *Phoma* and *Curvularia* have been isolated from environmental plastic particles^49^, while *Rhodotorula* species are known to colonise plastic catheters in hospitals^33^. However, this is the first report of their systematic co-occurrence and predominance in complex consortia on MP in anthropogenically disturbed soils. While some of these pathogens showed similar relative abundances in the soil, our DESeq2 analysis unveiled several opportunistic human pathogens that were significantly associated with MP (Fig. 5), such as *N. diffluens*, a cryptococcal yeast known to be able to cause subcutaneous pathologies in humans^50^, *Rh. mucilaginosa*, a plastic-affine yeast-like eurybiont and nosocomial pathogen capable of causing invasive and systemic symptoms^33,51^, *P. herbarum*, a dematiaceous, filamentous species causing a spectrum of mycoses^52^, and *R. anthropophila*, a rather unknown melanised mould that inhabits the human respiratory system^53^. Therefore, in accordance with our third hypothesis, we demonstrate for the first time that MP hosts and even accumulates fungal pathogens of plants, animals, and humans in terrestrial ecosystems.

Unveiled plastiphilic fungi appeared to be well adapted to the surface-exposed plastisphere in the topsoil. These species exhibit a saprotrophic life stage and can therefore survive and reproduce, decoupled from host interactions, forming robust sapronoses^54^. Remarkably, most species significantly being associated with plastics were melanised. Melanisation provides protection from UV radiation, enables thermotolerance and is a prerequisite for an invasive potential^55^. Among the plastiphilic fungi were species with trophic versatility. *Rh. mucilaginosa* and *N. diffluens* are oligotrophic, while members of the genera *Leptosphaerulina* and *Phoma* can degrade complex biopolymers, dyes, and plastics due to their ligninolytic enzymes^49,56^. Overall, these fungi may thus form tightly attached, highly resistant and durable biofilms in the plastisphere that exploit micronutrients deposited on the MP surface.

Mechanistically, there are many reasons for the observed prevalence of pathogens in the plastisphere, including the production of invasive structures (e.g., appressoria), biofilm formation, mucilage secretion and thigmotropism. SEM and CLSM analysis synergistically evidenced the full spectrum of fungal biofilm components^30^ in the plastisphere, including propagules, ECM, and hyphal bundles (Fig. 1, Supplementary Fig. 7). SEM micrographs further showed conidia adhering to the polymer surface by a sort of mucilage and even peripheral outgrowths of hyphae that appeared to be invasive. Solid hydrophobic surfaces, such as polystyrene films, are known to induce morphological differentiation and formation of invasive structures in pathogenic fungi^29^. Consequently, thigmotropism, although not directly analysed, could be a substantial aspect of the plastic-fungus interaction landscape. As the identified opportunists were mostly hyphomycetes, the release of hydrophobins, a molecular autapomorphy of filamentous fungi, may play a role in the attachment to plastics in situ. Under laboratory conditions, filamentous fungi were shown to accomplish adhesion to plastic surfaces through these small proteins by augmenting cell wall hydrophobicity^30^. Together, these factors indicate a biological as well as ecological predestination of pathogenic fungi to colonise plastics in terrestrial systems, so that pathogen attachment and accumulation as observed here most likely occur in diverse biomes and on a global scale.

Fungal infections are on the rise worldwide, with human disturbance of the natural environment being a major driver of their occurrence^38^. The present study is the first to demonstrate the direct impact of plastic pollution on the accumulation of soil-borne pathogens, which has several ecological and epidemiological implications. The aggregation of pathogenic fungi on plastic, an omnipresent and extremely persistent pollutant, effectively makes it a potent source of infection and could open new infection routes, increasing the risk of disease for wildlife, livestock, and humans. Indeed, infections of humans by plastic-associated fungi have been observed, with *F. oxysporum* causing keratitis epidemics^47^ and *Rh. mucilaginosa* causing fungemia in hospital patients^33^. Both fungi were found in this study among the dominant or enriched species on plastic waste. The impact of pathogen-infested plastic waste may be particularly critical in tropical regions. These systems receive massive influxes of plastic waste due to an underdeveloped waste management. Aggravatingly, fungal pathogens are most abundant in tropical and subtropical soils^35,54^. Thus, MPs in countries such as Kenya could contribute to the already extremely high fungal infection-related mortality and morbidity of the population. Due to the longevity of most plastic types and the fact that fungi can thrive and most likely proliferate within the plastisphere, the pathogen load on its surface is likely to increase over time and may result in an enhanced pathogen-carrying capacity of ecosystems. In this scenario, shifts in the structure and functionality of soil mycobiomes are likely to occur, eventually culminating in biodiversity losses, species extirpations and disruption of canonical ecosystem functioning. Plastic particles in the topsoil can be transported by the wind as a vector over long distances across core-matrix boundaries, which may result in habitat expansion of attached fungi. In this way, pathogens will become invasive in other habitats or directly transmitted to naive hosts. If colonised plastic fragments enter a long-range, fluctuating cycle of transport and fallout during the plastic cycle^57^, spill-over events and epidemics could increase in frequency, with MPs serving the function of artificial 'super-spreaders'.

Presumably, plastics can also facilitate transformation of non-pathogenic species by providing persistent and protective niches for horizontal gene and chromosome transfer from co-attached pathogenic species, like it has been shown for *F. oxysporum* and non-pathogenic *Fusarium* species on other substrates^58^ and for bacteria on plastic in aquatic systems^22^. Densely packed biofilms of pathogens on plastic may even promote interspecific hybridisation between different attached pathogens, resulting in hybrid offspring with an altered pathogenic phenotype^59^. Xenobiotics and ions leaching from the plastic surface may additionally affect horizontal gene acquisition by facilitating uptake of exogenous DNA or cause mutations by directly acting on fungal DNA. The evolution of the invasive potential of opportunists is decoupled from the host and is instead related to their extremotolerant ecology^46^. Plastic as a selective, anthropogenic microhabitat could therefore act on the invasive potential of opportunists by selecting genes encoding for virulence factors like biofilm formation, melanisation and oligotrophism. There is evidence that genes and gene clusters that are present throughout a variety of phylogenetically diverse fungal pathogens are essential for both the establishment of infection in host epithelia and adhesion to plastic^60^. If plastisphere colonisation confers fitness advantages to certain opportunistic species in the soil through the provision of nutrients, stimulation of spore germination and propagation, enhanced stress resistance and dispersal, such genes could be selected. Ultimately, plastic pollution may thus contribute to the emergence of more virulent plastiphilic opportunistic pathogens. In any case, our findings demonstrate the need for further investigations to unravel, whether and to what extent plastic pollution contributes to the emergence of fungal infectious diseases and to elucidate the impact of plastic on the evolutionary processes of fungal pathogens.

## Conclusion

Our study reveals that MPs are selective micro-habitats for diverse fungal organisms in terrestrial systems, attracting communities distinct from the surrounding soil. The finding of a core mycobiome in the plastisphere suggests that some fungi are better adapted to life on plastic surfaces than others and that systematic colonisation might be conceivable. Consequently, plastic debris may lead to the emergence of non-natural fungal communities in soils all over the world. Furthermore, we demonstrate that MP hosts and even accumulates fungal pathogens of plants and animals in terrestrial systems in general, the immediate human environment, and sub-Saharan Africa in particular. Here, plastisphere mycobiomes were primarily characterised by opportunistic human pathogens of the classes Dothideomycetes and Tremellomycetes, including *Phoma*-like filamentous fungi and a cryptococcal yeast. These findings implicate that these ubiquitous, extremely persistent pollutants are potential direct sources of infection and could open new infection routes, e.g., through increased pathogen loads and pathogen vectoring, possibly increasing the risk of disease in wildlife and humans. Future studies should analyse the ecological as well as epidemiological consequences of these most likely global phenomena, while policy makers should consider classifying plastic debris as a potential threat to human health.

## Material and methods

### Sampling

On March 9 and 14 2019, samples were collected from the topsoil of five distinct dumping sites within the municipal boundaries of Siaya (Siaya County, Western Kenya) (Supplementary Fig. 1). Site 1 was a landfill in Siaya central (Landfill 1; 0°3′50.04″N, 34°16′54.479″ E), site 2 was a roadside in the Mbaga village (roadside; 0°3′24.84″ N, 34°18′19.8″ E), site 3 was the Ramba marketplace (Market; 0°3′24.84″ N, 34°16′29.999″ E), site 4 was a courtyard at Aringo Estate (Courtyard; 0°4′45.48″ N, 34°16′48.359″ E) and site 5 was a second landfill in Siaya central (Landfill 2; 0°3′49.32″ N, 34°16′54.12″ E). Soil samples were taken from five randomly chosen spots within each location, using a sterile metal spoon to scrape the samples directly from the topsoil layer, adding up to a total of 25 samples. Georeferencing and image acquisition of the samples taken were carried out immediately on site using the *DiversityMobile* application^62^. All data was uploaded to the *DiversityCollection*^61^ database module via the application to ensure backup and easy access as well as traceability and reusability^41,62^. Upon collection in the field, the sample containers were immediately sealed in plastic boxes along with silica gel sachets to dry the samples and inhibit fungal growth. In the laboratory, samples were put on ice to perform selective subsampling.

### Selective subsampling

First, all visible non-soil fragments were selected and separated from the main sample using sterile tweezers. For each sample, 100 mg of soil material was collected with a sterile spatula into a screw-cap tube using a fine balance to generate a soil subsample. Meanwhile, the separated fragments were visually characterised under a Stemi SV 11 stereo microscope (Zeiss, Oberkochen, Germany) to distinguish plastic particles. To generate a corresponding plastic subsample, 100 mg of small plastic particles were thoroughly washed twice with sterile dH_2_O to remove attached soil particles and then placed in a tube (larger fragments were intentionally disregarded). To determine the size distribution, 100 plastic fragments per site and sample were randomly measured under the stereo microscope. The particle size ranged from ca. 3–30 mm, with the majority (approx. 78%) of particles < 5 mm. Thus, each plastic subsample was considered a MP subsample^4^. All 50 subsamples were stored at − 20 °C until further processing.

### DNA extraction

Whole metagenomic DNA extraction from both soil and plastic subsamples was performed using the NucleoSpin^®^ Soil kit (MACHEREY NAGEL, Düren, Germany) following the manufacturer’s specifications, after initial adjustments for all subsamples in parallel. Briefly, the samples were homogenised applying a FastPrep FP120 (ThermoSavant, Biogene, United States) cell disrupter and cells were lysed using the SL1 lysis buffer supplied with the kit. Optimisation of cell lysis conditions and DNA yields was achieved by adding 100 μl of Enhancer SX (provided by the NucleoSpin^®^ Soil kit) to the cell lysis reaction. DNA content was recorded and quality-checked using the NanoDrop^®^ ND-1000 UV–Vis Spectrophotometer (Thermo Fisher Scientific, Waltham, United States).

### ITS amplicon library preparation and sequencing

Dual-indexed ITS1 and 2 amplicons were generated via a two-step PCR processing during library preparation, following the operational design described in Guerreiro et al.^63^. In the first PCR step, four modified versions of the forward primer ITS1F (5ʹ-CTTGGTCATTTAGAGGAAGTAA-3ʹ) and the reverse primer ITS4 (5ʹ-TCCTCCGCTTATTGATATGC-3ʹ), were applied, each containing a unique ‘TAG’ region and a part of the Illumina sequencing primer. The first PCR step was carried out using 5.5 μl GoTaq^®^ Colourless Master Mix (Promega, Fitchburg, United States), 6.5 μl sterile PCR grade dH_2_O, 1 µl of each primer [10 μM] and 1 μl DNA template (1:100 dilution of the metagenomic DNA extract in PCR grade dH_2_O), adding to a final volume of 14.5 μl. Thermal cycling was conducted in a Primus 96 Thermal Cycler (MWG-Biotech, Ebersberg, Germany) consisted of an initial heat-activation at 95 °C for 3 min, followed by 33 cycles of a denaturation step at 94 °C for 27 s, an annealing step at 57 °C for 1 min and an elongation step at 72 °C for 1:30 min. Each PCR program ended with a final elongation at 72 °C for 7 min. The remaining reactants were cleaned up enzymatically during an ExoSAP digestion (Exonuclease I and Shrimp Alkaline Phosphatase, New England Biolabs, Ipswich, US), at 37 °C for 15 min, followed by a heat inactivation of the enzymes. The second PCR step was run under the same conditions as the first, with a higher total reaction volume of 25 μl, only 5 cycles and a primer combination consisting of the Illumina sequencing primer (dnatech.genomecenter.ucdavis.edu), an INDEX sequence, for the index-run and P5 and P7 adapter sequences, respectively. Combination of the TAG (PCR1) and INDEX (PCR2) sequences allowed the creation of unique sequence patterns for each environmental sample for the multiplexing of amplicons. Successful amplicon generation was confirmed by agarose gel electrophoresis (0.8% w/v, 0.5× TBE buffer at 75 V) and subsequent imaging of the DNA stained with ethidium bromide under UV light exposure (GelDocTM Station, Invitrogen, Carlsbad, United States). Resulting amplicons were pooled to equimolar concentrations. The amplicons were sequenced at the Genomics Service (LMU Biocenter, Munich) on an Illumina MiSeq (Illumina, Inc., San Diego, USA) with v3 chemistry (2 × 300 bp paired-end sequencing).

### Sequence data processing

From the obtained raw reads, barcodes were removed, and the reads were demultiplexed using extract_barcodes.py and split_libraries_fastq.py as implemented in QIIME^64^. The demultiplexed reads were then imported to the QIIME2 pipeline^65^ and remaining adapter and primer sequences were clipped using the tool cutadapt^66^. Quality filtering, including removal of chimeric sequences and dereplication of exact sequence variants was performed using the DADA2 plugin under QIIME2^64^, with a maximum expected error of 2 and a minimum fold-abundance of parent sequences of 1 over potential chimeras. OTUs were clustered from the amplicon sequence variants (ASVs) from the DADA2 output using VSEARCH and a 97% sequence similarity threshold. Taxonomy assignment was performed using the QIIME2-implemented command feature-classifier classify-sklearn and a Naive-Bayesian classifier, previously trained on the UNITE dynamic database (v.8.0)^67^ with a chunk size of 20,000. OTU representative sequences were then taxonomically classified using a 97% sequence similarity threshold and a minimum confidence score of 70%.

### Statistics and data analyses

Samples with less than 1000 contained sequences^68^ and OTUs showing less than 100 reads and/or occurrence in only one sample were removed from the dataset. Alpha diversity analysis included species richness (Observed, Chao1), Pielou’s (species) evenness and Shannon diversity and was based on OTU abundance data rarefied to the lowest common sequence number (2496 sequences). One-way ANOVA was applied to test for significant differences in all four indices between substrates and sites. For beta diversity analyses, square root transformed OTU counts of the non-rarefied dataset and taxa subsets (plant pathogens, animal pathogens, saprotrophs) were used to compile (distance) resemblance matrices based on Bray–Curtis (dis)similarity metric (k = 3). Non-metrical multidimensional scaling (nMDS) ordination was used to visualise compositional differences between epiplastic and soil mycobiomes. Main and pairwise permutational multivariate analyses of variance (PERMANOVA) were applied to test for statistically significant variance in fungal community composition between sites and substrates based on 9999 permutations at a significance level of p < 0.05. Homogeneity of dispersion (PERMDISP)^69^ was carried out based on calculated distances to centroids to assess whether significant PERMANOVA results were based on location or dispersion effects (9,999 permutations at a significance level of p < 0.05). To identify taxa that discriminated plastisphere and soil communities, DESeq2 analysis^70^ was performed as implemented in RStudio considering only OTUs with a relative abundance of ≥ 0.1% across all samples. Alpha and beta diversity analyses, differential abundance testing, and data visualization were conducted using the RStudio packages vegan^71^, phyloseq^72^ and ggplot^73^. ANOSIM, PERMANOVA, PERMDISP and SIMPER analysis were performed with the Primer 7 software package and the add-on package PERMANOVA + (PRIMER-e Ltd, Plymouth, United Kingdom)^74^.

### Phylogenetic analysis

To visualise the evolutionary distance of the core fungal taxa of the plastisphere, a phylogenetic tree was compiled. Core fungal (PCM) taxa were defined as those phylotypes accounting for ≥ 0.5% of all plastisphere reads and contributing with ≥ 0.3% to the dissimilarity between soil-dwelling and plastic-associated communities based on SIMPER analysis, while occurring in plastic samples across all five sites. First, nucleotide sequences were aligned using MUSCLE^75^ as implemented in MEGA-X^76^. Phylogenetic relationships were deduced by Maximum Likelihood method, using Tamura-Nei distance^77^ as a substitution model and bootstrap method based on 1000 replications as a phylogeny test. The tree was calculated in MEGA-X^76^ and visualised using iTOL version 4^78^. Trophic modes, host ranges and human pathogenicity potential were inferred by querying genera and species with various fungal and biological databases, including FUNGuild^79^, Encyclopedia of Life^80^, Index Fungorum (http://www.indexfungorum.org) and USDA^81^ as well as appropriate clinical and scientific literature. For each taxon only probable and highly probable matches were considered.

### Scanning electron microscopy

Eight MP fragments were randomly selected from samples of each site and preserved for SEM. The fragments were washed with fresh ddH_2_O and dried for 12 h at 70 °C. Dry samples were sputter-coated using a S150A Sputter Coater (Edwards, Irvine, USA) with a gold layer of 2 nm. The coated plastic fragments were visualised and imaged using a Quanta 200 electron microscope (FEI, Hillsboro, United States) with a 10 kV electron beam and varying magnifications.

### Confocal scanning laser microscopy

For staining, MP fragments were added to 20 µL of a solution containing 10 µg/mL of a Concanavalin A-Alexa Fluor™ 633 conjugate and 5 µM Syto™ 9 (Invitrogen, Carlsbad, United States) and incubated 20 min in the dark. ConA is a widely used lectin that selectively binds to glucose and mannose residues present in the cell wall of a variety of fungi^82^, while the conjugated Alexa dye shows far red fluorescence when stimulated with red light. SytoTM 9 is an excellent green-fluorescent nuclear and chromosomal counterstain for both prokaryotes and eukaryotes^83^. Afterwards, they were washed three times in sterile tap water and CLSM was carried out using a Leica SPE confocal microscope (Leica Microsystems, Wetzlar, Germany) with a 10× magnification lens. Blind deconvolution was applied to all 3D image stacks using the Auto-QuantTM deconvolution algorithm implemented in the LASX software. 3D image Z stacks of single fluorophores were merged into RGB images using the FIJI software^84^. GIMP software was used to augment brightness and enhance contrast by linear histogram stretching. To preserve comparability, relevant settings were adjusted equally in shown images.

## Supplementary Information

Supplementary Information 1.

Supplementary Information 2.

## Data Availability

Sequences from community barcoding are linked under BioProject accession number PRJNA705067. Environmental samples are stored in the collection at the Mycology Department, University of Bayreuth. Further data cited in the manuscript are available as Supplementary Data. All other relevant data is available upon request.
