# Supplementary material for: Microplastics accumulate fungal pathogens in terrestrial ecosystems
Source: Sci Rep. 2021 Jul 15;11:13214. doi: 10.1038/s41598-021-92405-7 (PMC8282651; doi:10.1038/s41598-021-92405-7)
Supplement: Supplementary file 1 — Supplementary Information 1. [file 41598_2021_92405_MOESM1_ESM.docx]

**Supplementary Information**

Gkoutselis et al. *Microplastics Accumulate Fungal Pathogens in Terrestrial Ecosystems.*


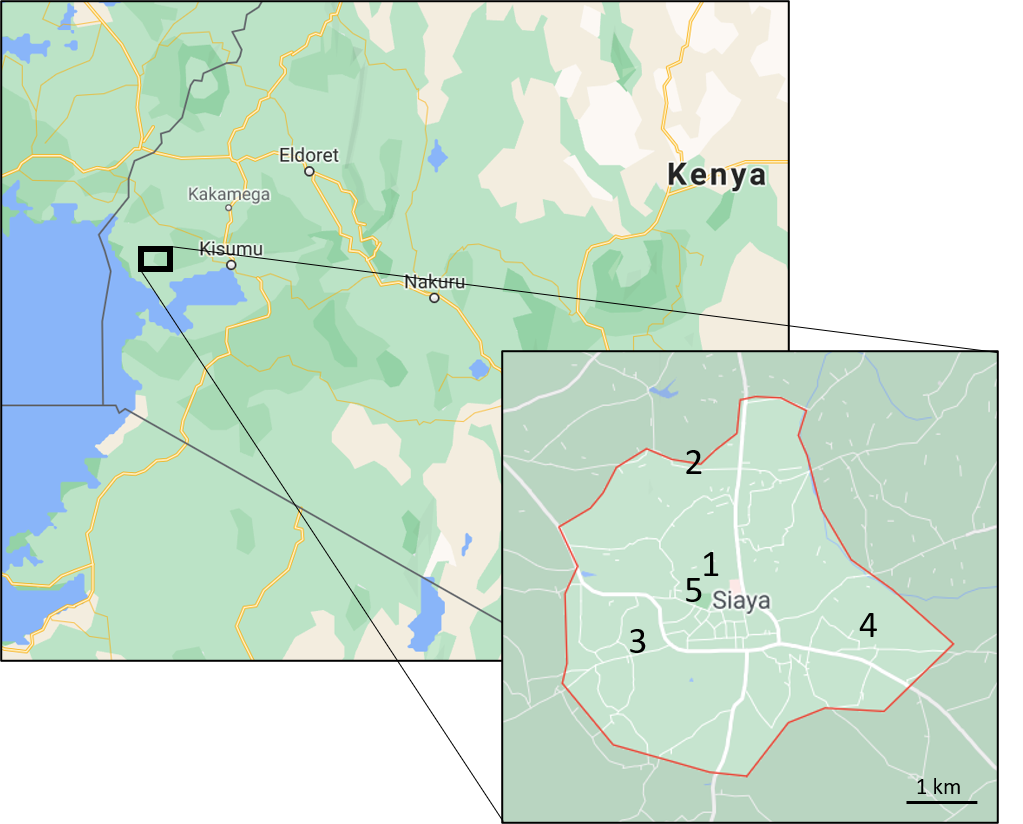


**Supplementary Figure 1.** Map section of Western Kenya and bordering countries, with small box highlighting the study area (Top) and zoom-in showing the sampling sites 1-5 within the municipal boundary of Siaya (Bottom).


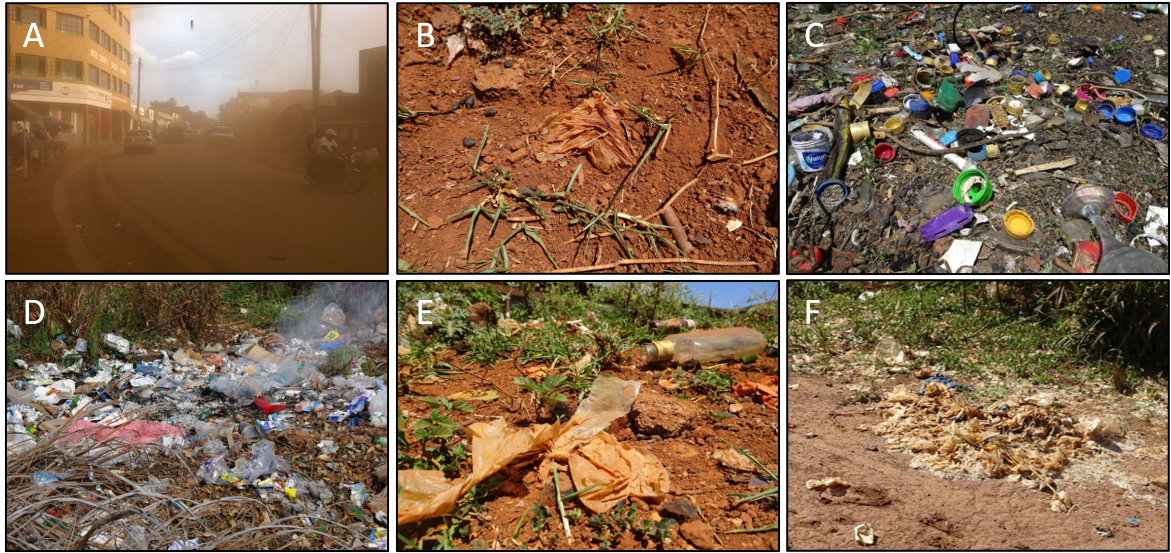


**Supplementary Figure 2.** Urban scenery in Siaya during the dry season and plastic waste accumulations at the five sampling sites. (A) Road in the centre of Siaya; (B) (Ramba) Market; (C) Landfill 1 (Siaya centre); (D) (Siaya centre) Landfill 2; (E) Courtyard (Aringo estate); (F) Roadside.


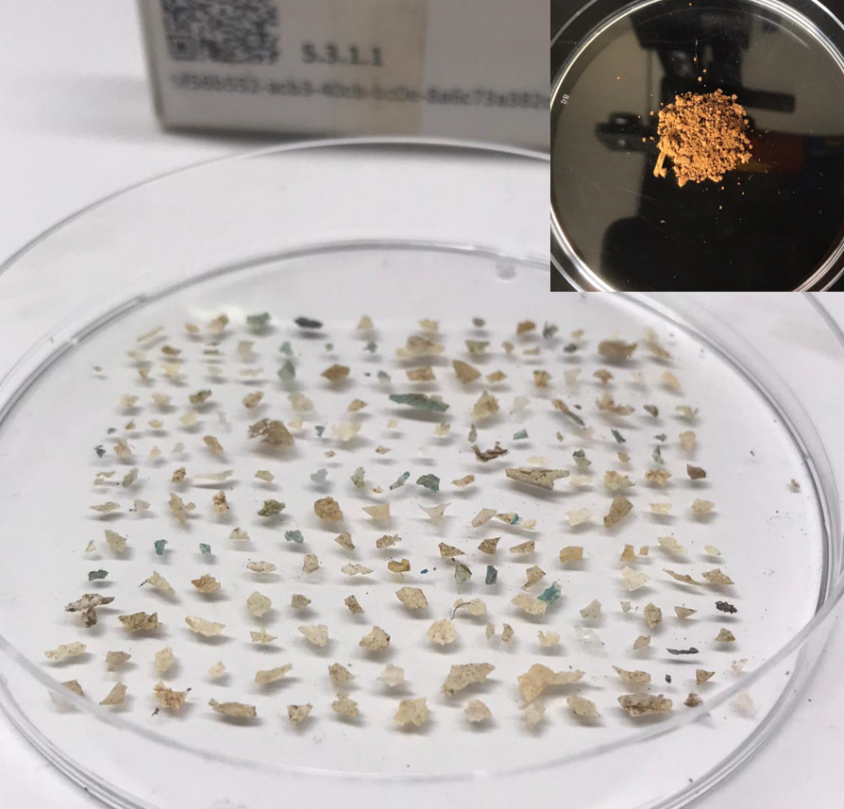


**Supplementary Figure 3.** Exemplary plastic fragments subsampled from one of the environmental samples as used in this study for SEM, CLSM and ITS metabarcoding. Fragment size ranged from approximately 1-30 mm with the majority of particles < 5 mm (78%; n = 500). Soil subsample generated from the same environmental sample in the inset.


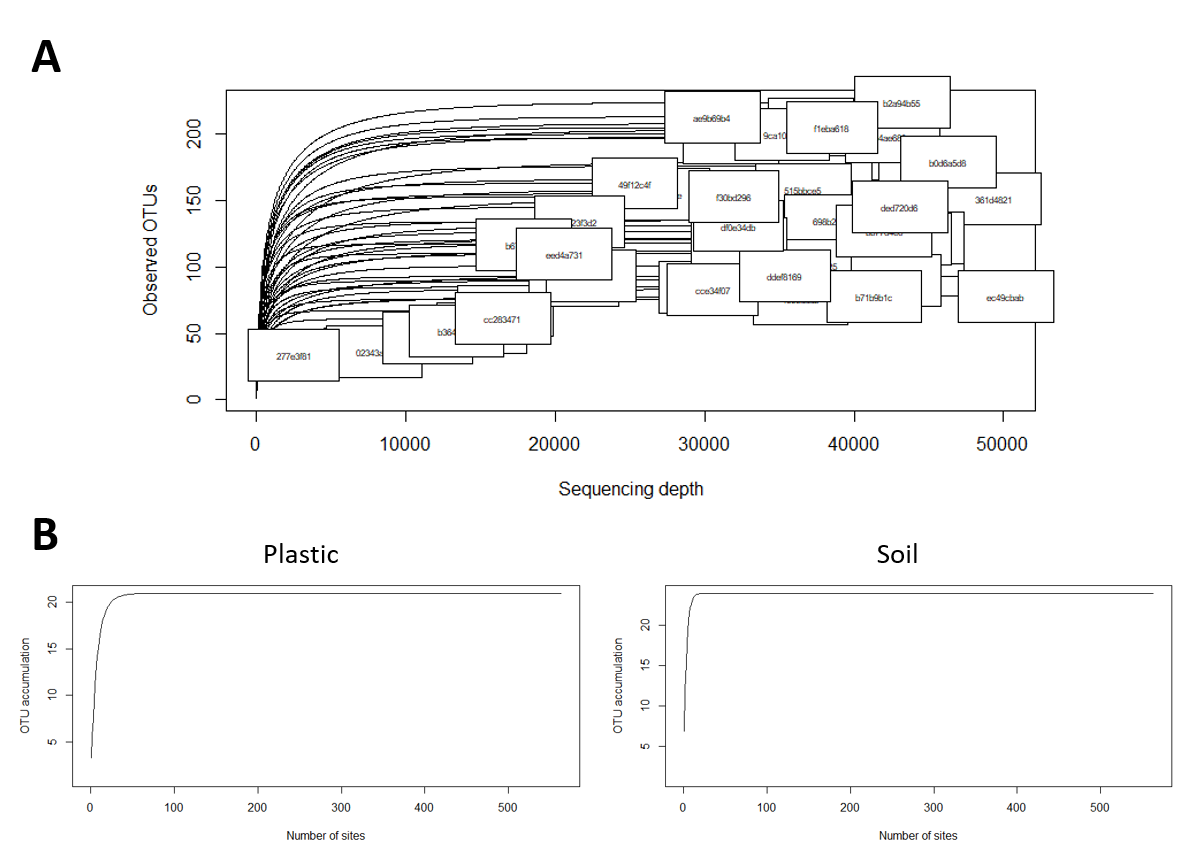


**Supplementary Figure 4.** Rarefaction (A) and species accumulation (B) curves of all samples analysed in this study. Rarefaction and species accumulation curves were obtained using the rarecurve and specaccum functions, respectively, in the package vegan^1^.


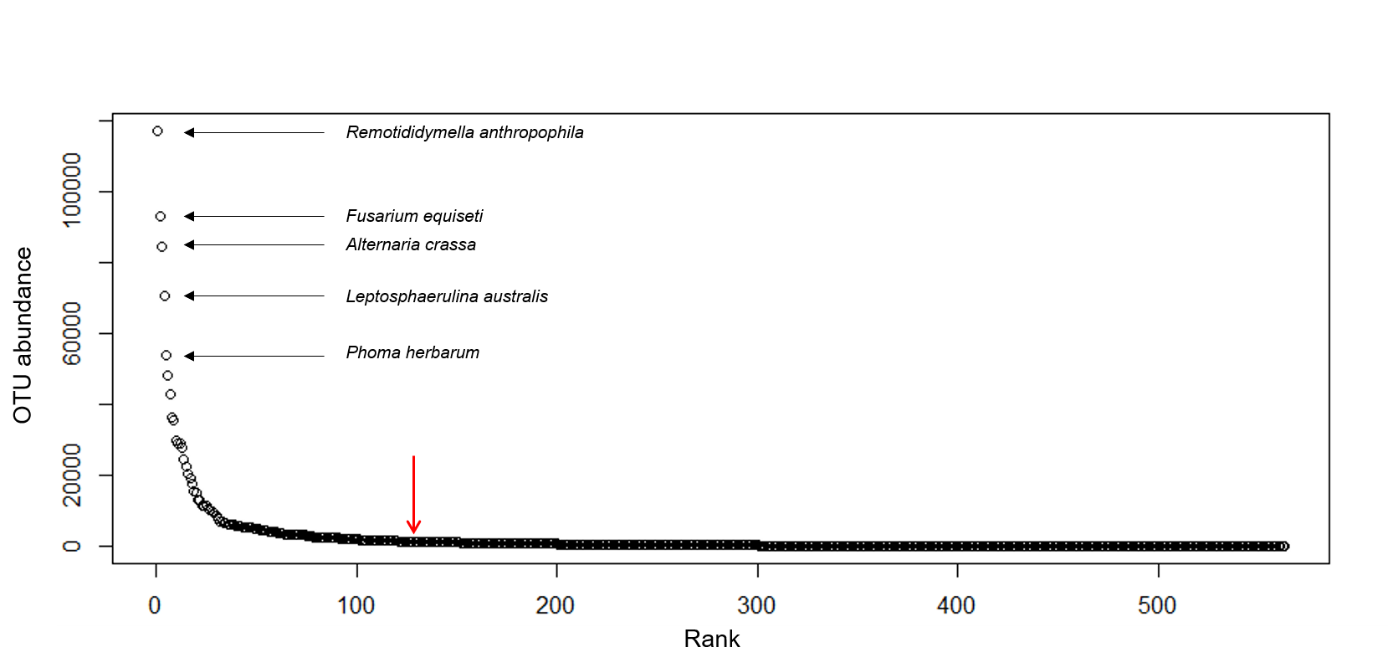


**Supplementary Figure 5.** Rank abundance curve for fungal ITS sequence data from both substrate types combined. OTUs were ordered on the x-axis from most to least abundant. 128 OTUs showed a relative abundance > 0.1% (indicated by the red arrow). The top five most abundant fungal phylotypes were *Remotididymella anthropophila* (8.1%), *Fusarium equiseti* (6.4%), *Alternaria crassa* (5.8%), *Leptosphaerulina australis* (4.9%) and *Phoma herbarum* (3.7%).


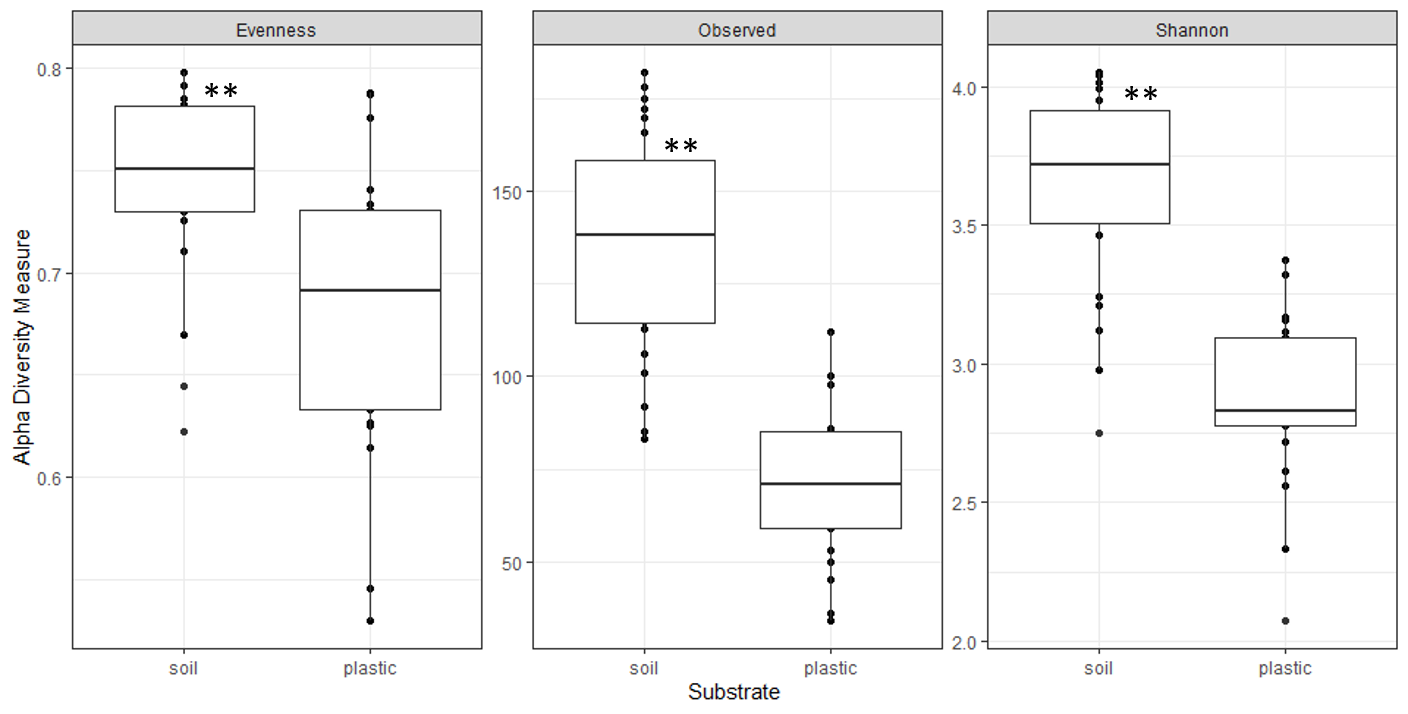


**Supplementary Figure 6.** Estimated (Pielou’s) species evenness, richness (Observed) and diversity (Shannon) on rarefied OTU counts according to substrates. Data were rarefied to the maximum reads of the smallest sample. (**) ≤ 0.01.


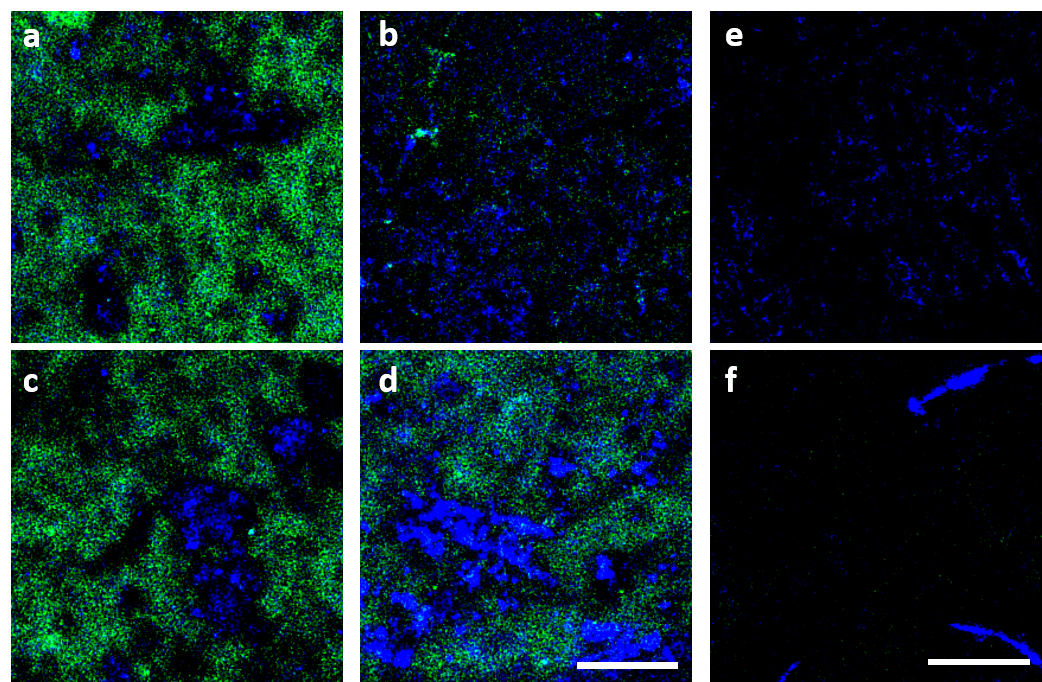


**Supplementary Figure 7.** Fungal biofilm formation visualised by CLSM. **a-d** Localised and congregated fungal ECM patches (blue) surrounded by massive amounts of nucleic acids (green) after staining with Concavalin A and Syto 9, respectively. **e,f** Unstained MP particles with signs of autofluorescence.


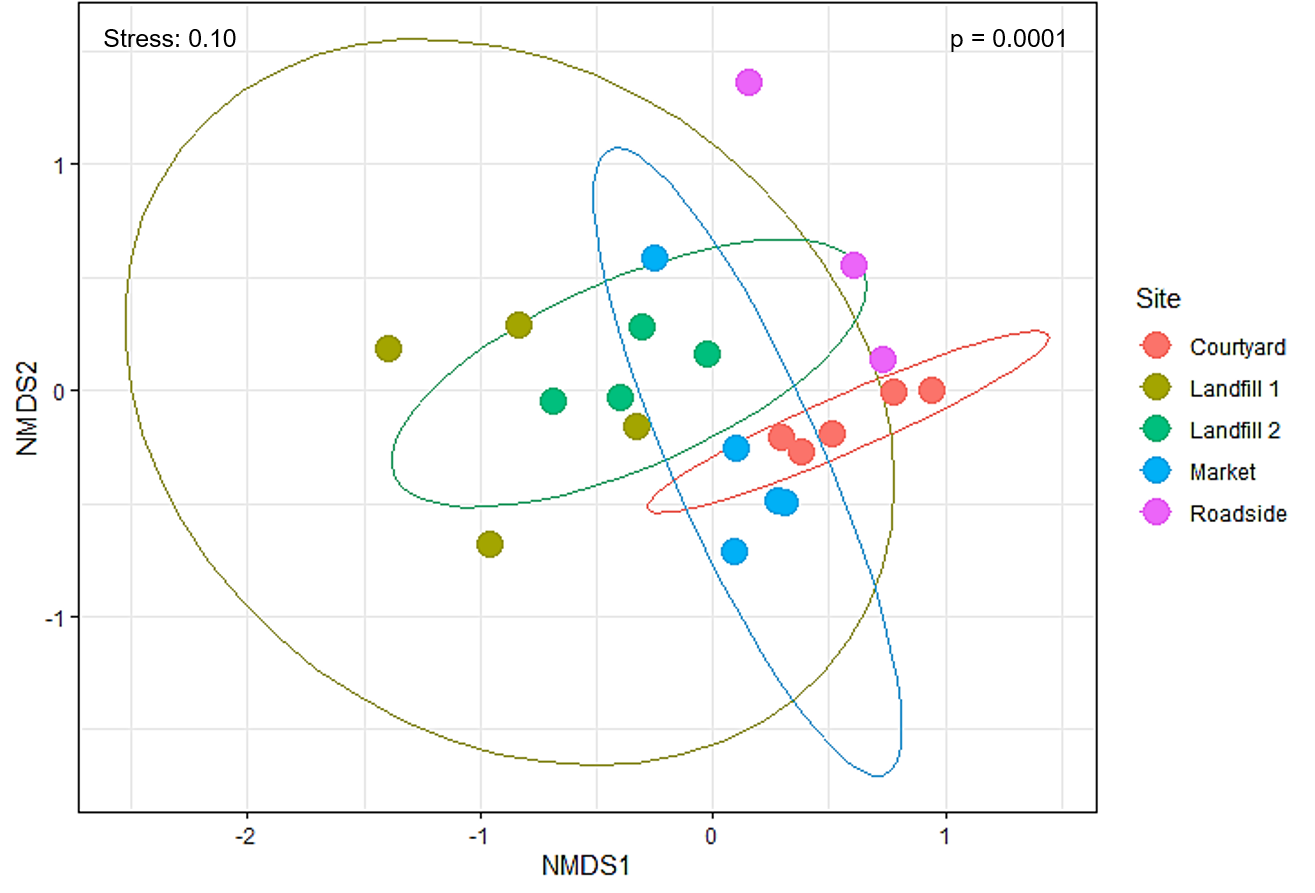


**Supplementary Fig. 8.** Variation in plastisphere community composition among sites visualised by NMDS. The plot is based on Bray-Curtis dissimilarity matrix of square root transformed relative abundances of OTUs. Ellipsoids represent a 95% confidence interval surrounding the data points of each factor group. Significant variation between substrates was tested by PERMANOVA (included p-value) (Supplementary Table 5). NMDS ordination stress value included.

**Supplementary Table 1.** Univariate diversity indices of soil and plastisphere mycobiomes calculated on read counts of OTUs rarefied at the smallest library size (2,496 reads).

| **Sample ID** | **Site** | **Substrate** | **Observed** | **Chao1** | **Shannon** | **Pielou** |
| --- | --- | --- | --- | --- | --- | --- |
| 692fb42a | Courtyard | plastic | 80 | 83.6 | 3.056107 | 0.6974187 |
| 7584931a | Courtyard | plastic | 65 | 65.42857 | 2.614537 | 0.6263283 |
| 85ddf6d7 | Courtyard | plastic | 84 | 85.25 | 3.112425 | 0.7024494 |
| bb77d4e6 | Courtyard | plastic | 112 | 132.3125 | 3.321699 | 0.7039736 |
| ddef8169 | Courtyard | plastic | 69 | 80.375 | 3.091433 | 0.7301264 |
| 59ae7426 | Courtyard | soil | 170 | 203.44444 | 3.824267 | 0.7446296 |
| 5a30b8a6 | Courtyard | soil | 83 | 104.08333 | 2.748608 | 0.6220203 |
| 6ff660a9 | Courtyard | soil | 172 | 199.85714 | 4.039253 | 0.7847028 |
| 9ca10b1d | Courtyard | soil | 166 | 181.0303 | 4.011547 | 0.7847332 |
| f1eba618 | Courtyard | soil | 178 | 243.04545 | 4.049234 | 0.7814364 |
| 02343a00 | Landfill 1 | plastic | 36 | 36 | 2.822582 | 0.7876565 |
| 28f1e6b1 | Landfill 1 | plastic | 53 | 53.6 | 2.775277 | 0.6990108 |
| 591f156d | Landfill 1 | plastic | 45 | 45 | 2.952523 | 0.7756198 |
| cce34f07 | Landfill 1 | plastic | 72 | 80.75 | 2.334371 | 0.5458389 |
| 1d232c7a | Landfill 1 | soil | 92 | 105 | 3.211911 | 0.7103187 |
| 8a23f3d2 | Landfill 1 | soil | 123 | 151.875 | 3.808204 | 0.7913671 |
| b674a230 | Landfill 1 | soil | 113 | 120.33333 | 3.593112 | 0.7600629 |
| ded720d6 | Landfill 1 | soil | 133 | 168.76923 | 3.900984 | 0.7976903 |
| 4355aaab | Landfill 2 | plastic | 71 | 71.6 | 3.157301 | 0.7406844 |
| b3640270 | Landfill 2 | plastic | 50 | 56 | 2.071802 | 0.5295985 |
| b71b9b1c | Landfill 2 | plastic | 60 | 69.16667 | 2.558974 | 0.6250022 |
| ec49cbab | Landfill 2 | plastic | 64 | 81 | 2.715528 | 0.6529465 |
| 2a66c0b5 | Landfill 2 | soil | 141 | 178.05882 | 3.885218 | 0.7850892 |
| 338fe04c | Landfill 2 | soil | 85 | 90 | 3.243631 | 0.7301115 |
| 361d4821 | Landfill 2 | soil | 106 | 128.14286 | 3.122082 | 0.6694806 |
| 698b2a32 | Landfill 2 | soil | 115 | 125 | 3.461422 | 0.7294987 |
| df0e34db | Landfill 2 | soil | 101 | 116.8125 | 2.974792 | 0.6445752 |
| 5e479d0f | Market | plastic | 100 | 111.76923 | 2.830259 | 0.6145829 |
| 69e25525 | Market | plastic | 86 | 105.125 | 2.819617 | 0.6330035 |
| 8fe6aadc | Market | plastic | 73 | 79.11111 | 2.952406 | 0.6881328 |
| cc283471 | Market | plastic | 59 | 59.75 | 2.814548 | 0.6902567 |
| eed4a731 | Market | plastic | 100 | 106 | 3.376155 | 0.7331228 |
| 42c1a08d | Market | soil | 140 | 166.4 | 3.612922 | 0.7311176 |
| 515bbce5 | Market | soil | 115 | 136.9375 | 3.517081 | 0.741229 |
| 64ae683e | Market | soil | 156 | 167 | 3.949338 | 0.7820694 |
| ae9b69b4 | Market | soil | 175 | 220.04762 | 3.993397 | 0.773197 |
| b2a94b55 | Market | soil | 182 | 215.11538 | 3.951516 | 0.7593219 |
| 277e3f81 | Roadside | plastic | 34 | 34 | 2.776126 | 0.7872496 |
| afa51847 | Roadside | plastic | 85 | 92.58333 | 2.830075 | 0.6370239 |
| bbb0d5b2 | Roadside | plastic | 98 | 107.06667 | 3.16886 | 0.6911412 |
| 1f31613e | Roadside | soil | 136 | 146 | 3.681321 | 0.7493547 |
| 49f12c4f | Roadside | soil | 141 | 166.07143 | 3.703314 | 0.7483316 |
| 632c1492 | Roadside | soil | 145 | 174.29167 | 3.740348 | 0.7515668 |
| b0d6a5d8 | Roadside | soil | 129 | 149.77778 | 3.52438 | 0.7252091 |
| f30bd296 | Roadside | soil | 141 | 164.4 | 3.72844 | 0.7534089 |

**Supplementary Table 2.** One-way ANOVA test for significant differences in alpha diversity between substrate types (soil and plastic). d.f.: degrees of freedom, Sq: squares, crit. F-value: critical F-value. Significant results (p < 0.05) are highlighted in bold.

|  |  | **d.f.** | **sum Sq** | **mean Sq** | **test F** | **p-value** | **crit. F-value** |
| --- | --- | --- | --- | --- | --- | --- | --- |
|  | **Between** | 1 | 45415.6 | 45415.6 | 66.1 | **3.1272E-10** | 4.1 |
| **Observed** | **Within** | 43 | 29561.6 | 687.5 |  |  |  |
|  | **Total** | 44 | 74977.2 |  |  |  |  |
|  | **Between** | 1 | 74091.6 | 74091.6 | 64.9 | **3.9758E-10** | 4.1 |
| **Chao1** | **Within** | 43 | 49115.3 | 1142.2 |  |  |  |
|  | **Total** | 44 | 123206.9 |  |  |  |  |
|  | **Between** | 1 | 6.7 | 6.7 | 59.4 | **1.2308E-09** | 4.1 |
| **Shannon** | **Within** | 43 | 4.8 | 0.1 |  |  |  |
|  | **Total** | 44 | 11.5 |  |  |  |  |
|  | **Between** | 1 | 0.0 | 0.0 | 13.2 | **0.0007** | 4.1 |
| **Pielou** | **Within** | 43 | 0.1 | 0.0 |  |  |  |
|  | **Total** | 44 | 0.2 |  |  |  |  |

**Supplementary Table 3.** One-way ANOVA test for significant differences in alpha diversity between sites for the plastisphere mycobiome. d.f.: degrees of freedom. Sq: squares. crit. F-value: critical F-value.

|  |  | **d.f.** | **sum Sq** | **mean Sq** | **test F** | **p-value** | **crit. F-value** |
| --- | --- | --- | --- | --- | --- | --- | --- |
|  | **Between** | 4 | 3304.2 | 826.0 | 2.3 | 0.1082 | 3.0 |
| **Observed** | **Within** | 16 | 5851.6 | 365.7 |  |  |  |
|  | **Total** | 20 | 9155.8 |  |  |  |  |
|  | **Between** | 4 | 4306.8 | 1076.7 | 1.9 | 0.1552 | 3.0 |
| **Chao1** | **Within** | 16 | 8946.2 | 559.1 |  |  |  |
|  | **Total** | 20 | 13253.0 |  |  |  |  |
|  | **Between** | 4 | 0.5 | 0.1 | 1.5 | 0.2574 | 3.0 |
| **Shannon** | **Within** | 16 | 1.4 | 0.1 |  |  |  |
|  | **Total** | 20 | 1.9 |  |  |  |  |
|  | **Between** | 4 | 0.0 | 0.0 | 0.6 | 0.6905 | 3.0 |
| **Pielou** | **Within** | 16 | 0.1 | 0.0 |  |  |  |
|  | **Total** | 20 | 0.1 |  |  |  |  |

**Supplementary Table 4.** PERMANOVA main test of fungal communities on different substrate types based on Bray–Curtis similarity of OTUs. p-values were obtained from type III sums and 9999 permutations under the full model. d.f.: degrees of freedom. Sq: squares. perms: number of unique permutations per comparison. Significant results (p < 0.05) are highlighted in bold.

|  |  | **d.f.** | **sum Sq** | **mean Sq** | **Pseudo-F** | **p-value** | **perms** |
| --- | --- | --- | --- | --- | --- | --- | --- |
|  | **Location** | 1 | 6786.1 | 6786.1 | 2.9916 | **0.0002** | 9884 |
| **All fungi** | **Residuals** | 43 | 97539 | 2268.3 |  |  |  |
|  | **Total** | 44 | 1.04E+05 |  |  |  |  |
| **Plant pathogens** | **Location** | 1 | 3892.8 | 3892.8 | 21.886 | **0.0107** | 9902 |
|  | **Residuals** | 43 | 76482 | 1778.7 |  |  |  |
|  | **Total** | 44 | 80375 |  |  |  |  |
| **Animal pathogens** | **Location** | 1 | 6640.8 | 6640.8 | 41.323 | **0.0001** | 9930 |
|  | **Residuals** | 43 | 69103 | 1607.1 |  |  |  |
|  | **Total** | 44 | 75744 |  |  |  |  |
| **Sapro- trophic fungi** | **Location** | 1 | 7909.7 | 7909.7 | 27.623 | **0.0005** | 9899 |
|  | **Residuals** | 43 | 1.23E+09 | 2863.4 |  |  |  |
|  | **Total** | 44 | 1.31E+09 |  |  |  |  |

**Supplementary Table S5.** PERMANOVA main test of plastisphere fungal community on different sites based on Bray–Curtis similarity of OTUs. P-values were obtained from type III sums and 9999 permutations under the full model. d.f.: degrees of freedom. Sq: squares. perms: number of unique permutations per comparison. Significant results (p < 0.05) are highlighted in bold.

|  | **d.f.** | **sum Sq** | **mean Sq** | **Pseudo-F** | **p-value** | **perms** |
| --- | --- | --- | --- | --- | --- | --- |
| **Location** | 4 | 18486 | 4621.5 | 2.3525 | **0.0001** | 9833 |
| **Residuals** | 16 | 31432 | 1964.5 |  |  |  |
| **Total** | 20 | 49918 |  |  |  |  |

**Supplementary Table 6.** PERMANOVA and PERMDISP pairwise tests of plastisphere fungal communities from different sites based on Bray–Curtis similarity of OTUs. PERMANOVA P-values were obtained from type III sums and 9999 permutations under the full model. Significant results (p < 0.05) are highlighted in bold.

|  |  | **PERMANOVA** | | **PERMDISP** | |
| --- | --- | --- | --- | --- | --- |
| **Comparison** | | **t (perm)** | **p (perm)** | **t (perm)** | **p (perm)** |
| Landfill 1 vs. | Roadside | 1.3569 | **0.0266** | 0.41886 | 0.8037 |
|  | Landfill 2 | 1.2049 | 0.0834 | 2.1635 | 0.1212 |
|  | Market | 1.4619 | **0.0244** | 0.99944 | 0.4781 |
|  | Courtyard | 1.9735 | **0.0093** | 4.4602 | **0.0081** |
| Roadside vs. | Landfill 2 | 1.3893 | **0.0278** | 1.4637 | 0.2852 |
|  | Market | 1.3548 | 0.0542 | 0.62493 | 0.6782 |
|  | Courtyard | 1.4726 | **0.0184** | 3.465 | 0.6782 |
| Landfill 2 vs. | Market | 1.4428 | **0.0311** | 0.1454 | 0.9925 |
|  | Courtyard | 2.0345 | **0.0085** | 2.2477 | 0.0868 |
| Market vs. | Courtyard | 1.5713 | **0.0096** | 1.2809 | 0.5437 |

**Supplementary Table 7.** ANOSIM tests of fungal communities from different substrate types based on Bray–Curtis similarity of OTUs. Significant results (p < 0.05) are highlighted in bold.

|  | | **ANOSIM** | |
| --- | --- | --- | --- |
| **plastic vs. soil** | **R^2^** | | **p (perm)** |
| All fungi | 0.195 | | **0.0001** |
| Plant pathogens | 0.104 | | **0.005** |
| Animal pathogens | 0.235 | | **0.0001** |
| Saprotrophic fungi | 0.193 | | **0.0001** |

**Supplementary Table 8.** SIMPER analysis of plastisphere fungal communities contributing to the total similarity within and dissimilarity between different groups of sites. Aver. sim.: average percentage similarity within each group.

| **Aver. sim. [%]** | | **Average dissimilarity [%]** | | | | |
| --- | --- | --- | --- | --- | --- | --- |
| **Landfill 1** | 27.97 | **Landfill 1** | 81.37 | 70.07 | 74.84 | 78.13 |
| **Roadside** | 26.54 | **Roadside** |  | 75.13 | 73.78 | 68.55 |
| **Landfill 2** | 39.58 | **Landfill 2** |  |  | 68.19 | 71.36 |
| **Market** | 39.89 | **Market** |  |  |  | 63.35 |
| **Courtyard** | 50.9 |  | **Roadside** | **Landfill 2** | **Market** | **Courtyard** |

**Supplementary Table 9.** Fungal species of the plastisphere core mycobiome. Species IDs were ascertained from the official Index Fungorum website (http://www.indexfungorum.org). Ecological guild and opportunistic human pathogenicity were determined by analysing and interpreting data from the listed resources. Index Fung. ID: Index Fungorum ID. OHP: opportunistic human pathogenicity. AP: animal pathogen. PP: plant pathogen. EOL: Encyclopedia of Life. USDA: United States Department of Agriculture.

| **Taxon Name** | **Index Fung. ID** | **Ecol. Guild** | **OHP** | **References** | **Database Resources** |
| --- | --- | --- | --- | --- | --- |
| *Alternaria alternata* | 119834 | AP | + | 2 | EOL, FUNGuild, USDA |
| *Alternaria crassa* | 118901 | PP |  | 3 | USDA |
| *Cladosporium cladosporioides* | 294915 | AP | + | 2, 4 | EOL, FUNGuild, USDA |
| *Curvularia lunata* | 269889 | PP | + | 2 | EOL, FUNGuild, USDA |
| *Didymella glomerata* | 814105 | PP | + | 5 | EOL, USDA |
| *Epicoccum sorghinum* | 544157 | PP | + | 5 | EOL, USDA |
| *Fusarium delphinoides* | 512357 | AP | + | 6 | FUNGuild |
| *Fusarium equiseti* | 568603 | PP | + | 7 | FUNGuild, USDA |
| *Fusarium oxysporum* | 218547 | AP | + | 7 | EOL, FUNGuild, USDA |
| *Leptosphaerulina australis* | 333278 | PP | + | 8 | FUNGuild, USDA |
| *Myrothecium verrucaria* | 245274 | PP | + | 9 | FUNGuild, USDA |
| *Naganishia albida* | 813246 | AP | + | 10, 11, 12 |  |
| *Naganishia diffluens* | 813172 | AP | + | 13 |  |
| *Phoma herbarum* | 171008 | AP | + | 2, 5, 8 | EOL, FUNGuild, USDA |
| *Pseudopithomyces chartarum* | 551393 | PP | + | 11 | EOL, USDA |
| *Remotididymella anthropophila* | 819991 | AP | + | 14 |  |
| *Rhodotorula mucilaginosa* | 271749 | AP | + | 15, 16 | FUNGuild, USDA |
| *Stagonosporopsis cucurbitacearum* | 515660 | PP | + | 11 | EOL, USDA |

**Supplementary Table 10.** Cross-site occurrence of the fungal species found in the plastisphere. OTUs that returned the same species were collapsed into a representative one (species collapse). OTU ID refers to the first eight digits of the original 32-digit alphanumeric OTU UUID (see Supplementary Data 1).

|  | Site Number | Occurrence [reads] | | | | | |
| --- | --- | --- | --- | --- | --- | --- | --- |
| OTU ID | **Species** | **Overall** | **Landfill 1** | **Roadside** | **Market** | **Courtyard** | **Landfill 2** |
| 88e1e447 | *Aaosphaeria arxii* | 585 | 0 | 160 | 269 | 38 | 118 |
| 40e685e9 | *Acremonium antarcticum* | 321 | 149 | 25 | 118 | 0 | 29 |
| c9920a54 | *Acremonium furcatum* | 185 | 0 | 0 | 185 | 0 | 0 |
| 3e473508 | *Acremonium persicinum* | 0 | 0 | 0 | 0 | 0 | 0 |
| 8724cbf8 | *Acrocalymma medicaginis* | 553 | 0 | 335 | 136 | 82 | 0 |
| 64e9c3bd | *Acrocalymma vagum* | 14 | 8 | 6 | 0 | 0 | 0 |
| 100fbe66 | *Acrostalagmus annulatus* | 26 | 26 | 0 | 0 | 0 | 0 |
| 2b484c94 | *Alternaria alternata* | 10816 | 1216 | 1388 | 2164 | 800 | 5248 |
| db610013 | *Alternaria crassa* | 30672 | 385 | 73 | 4734 | 8151 | 17329 |
| 8aed07e2 | *Aphanoascus pinarensis* | 40 | 40 | 0 | 0 | 0 | 0 |
| fefc34e0 | *Aplosporella yalgorensis* | 78 | 0 | 0 | 67 | 0 | 11 |
| 96c37b52 | *Ascodesmis sphaerospora* | 0 | 0 | 0 | 0 | 0 | 0 |
| 947f1763 | *Aspergillus flavus* | 44 | 3 | 0 | 38 | 3 | 0 |
| a1ba5cd6 | *Aspergillus pseudodeflectus* | 362 | 229 | 0 | 0 | 0 | 133 |
| 085e5180 | *Aspergillus quadrilineatus* | 173 | 0 | 0 | 141 | 0 | 32 |
| a9be25c1 | *Aspergillus ruber* | 0 | 0 | 0 | 0 | 0 | 0 |
| 13f428e5 | *Aspergillus sydowii* | 0 | 0 | 0 | 0 | 0 | 0 |
| a8e5a95c | *Aspergillus tamarii* | 30 | 12 | 0 | 0 | 0 | 18 |
| 2842169a | *Aureobasidium subglaciale* | 342 | 13 | 16 | 0 | 257 | 56 |
| 79eaf25b | *Bipolaris cynodontis* | 2381 | 67 | 10 | 1427 | 832 | 45 |
| 6fbe76e9 | *Bipolaris gossypina* | 1535 | 0 | 221 | 53 | 1261 | 0 |
| b0980b00 | *Bipolaris sacchari* | 66 | 0 | 0 | 0 | 66 | 0 |
| 3bbd1a3d | *Calvatia rubroflava* | 80 | 0 | 0 | 0 | 0 | 80 |
| 5cc8d119 | *Cephaliophora tropica* | 57 | 57 | 0 | 0 | 0 | 0 |
| 93d4f54c | *Chaetomella raphigera* | 101 | 0 | 0 | 101 | 0 | 0 |
| 6d27f7f1 | *Chaetomium globosum* | 4585 | 4585 | 0 | 0 | 0 | 0 |
| 70b5280a | *Chaetomium homopilatum* | 16 | 16 | 0 | 0 | 0 | 0 |
| a391fd42 | *Chlorophyllum hortense* | 31 | 0 | 0 | 0 | 0 | 31 |
| 3e852cb9 | *Chroogomphus purpurascens* | 71 | 0 | 0 | 0 | 0 | 71 |
| 71f5057c | *Cladosporium austrohemisphaericum* | 46 | 0 | 19 | 7 | 20 | 0 |
| a9309203 | *Cladosporium cladosporioides* | 13944 | 963 | 5045 | 2925 | 2944 | 2067 |
| 139954f5 | *Cladosporium halotolerans* | 0 | 0 | 0 | 0 | 0 | 0 |
| e4815850 | *Clonostachys rogersoniana* | 1856 | 18 | 0 | 1814 | 24 | 0 |
| 71c8fe80 | *Clonostachys rosea* | 0 | 0 | 0 | 0 | 0 | 0 |
| 05c38622 | *Colletotrichum aenigma* | 100 | 80 | 0 | 0 | 6 | 14 |
| 543c657c | *Colletotrichum truncatum* | 135 | 0 | 0 | 101 | 0 | 34 |
| baa92a8c | *Coniochaeta fasciculata* | 0 | 0 | 0 | 0 | 0 | 0 |
| 5104531c | *Coprinopsis cinerea* | 778 | 49 | 0 | 656 | 0 | 73 |
| 1f98c3d8 | *Craterellus tubaeformis* | 295 | 295 | 0 | 0 | 0 | 0 |
| c57ad2a0 | *Cryptococcus dimennae* | 155 | 98 | 24 | 0 | 0 | 33 |
| 83d0f695 | *Ctenomyces serratus* | 47 | 0 | 0 | 35 | 12 | 0 |
| fcfce7b7 | *Curvularia brachyspora* | 149 | 0 | 25 | 26 | 26 | 72 |
| 07d31930 | *Curvularia coatesiae* | 290 | 0 | 73 | 58 | 49 | 110 |
| 56f7c912 | *Curvularia lunata* | 10952 | 413 | 155 | 2403 | 7821 | 160 |
| b412f62d | *Curvularia mebaldsii* | 107 | 40 | 30 | 0 | 0 | 37 |
| b0bee926 | *Curvularia nodulosa* | 67 | 0 | 0 | 67 | 0 | 0 |
| 370747a8 | *Curvularia prasadii* | 547 | 0 | 24 | 318 | 92 | 113 |
| 828bae04 | *Curvularia senegalensis* | 2177 | 134 | 52 | 126 | 1772 | 93 |
| a22222c7 | *Curvularia sporobolicola* | 1259 | 32 | 171 | 14 | 1042 | 0 |
| 21338f60 | *Cutaneotrichosporon terricola* | 15 | 0 | 0 | 15 | 0 | 0 |
| 3137920c | *Cyathus stercoreus* | 33 | 0 | 8 | 0 | 25 | 0 |
| 4197392a | *Cyphellophora gamsii* | 35 | 0 | 18 | 0 | 0 | 17 |
| c6ec10a7 | *Cyrenella elegans* | 494 | 0 | 0 | 16 | 447 | 31 |
| a478db37 | *Cystobasidium pinicola* | 98 | 80 | 0 | 0 | 18 | 0 |
| 56ce5e07 | *Cystobasidium slooffiae* | 98 | 0 | 0 | 21 | 77 | 0 |
| dc8537c6 | *Didymella exigua* | 800 | 258 | 50 | 271 | 87 | 134 |
| 65c51200 | *Didymella glomerata* | 20645 | 1148 | 3263 | 5416 | 9902 | 916 |
| e069fb7e | *Dothistroma pini* | 706 | 0 | 0 | 0 | 27 | 679 |
| 808af2fd | *Edenia gomezpompae* | 1847 | 96 | 149 | 561 | 817 | 224 |
| f430901a | *Epicoccum italicum* | 2271 | 918 | 157 | 356 | 379 | 461 |
| 6594f0e1 | *Epicoccum sorghinum* | 14348 | 947 | 1432 | 1534 | 10034 | 401 |
| e17fa01a | *Exophiala spinifera* | 98 | 0 | 0 | 0 | 98 | 0 |
| fc2ea086 | *Exserohilum rostratum* | 373 | 70 | 0 | 50 | 235 | 18 |
| c2b4df33 | *Funneliformis mosseae* | 0 | 0 | 0 | 0 | 0 | 0 |
| 99e0b776 | *Fusarium buharicum* | 361 | 0 | 0 | 299 | 62 | 0 |
| 91ca8c82 | *Fusarium delphinoides* | 6288 | 574 | 213 | 2847 | 2311 | 343 |
| cad7d4b9 | *Fusarium equiseti* | 29614 | 895 | 6600 | 9733 | 11357 | 1029 |
| 02e6db14 | *Fusarium kyushuense* | 83 | 39 | 0 | 0 | 0 | 44 |
| fa60eaa9 | *Fusarium nelsonii* | 63 | 0 | 63 | 0 | 0 | 0 |
| f422290d | *Fusarium oxysporum* | 7651 | 331 | 4750 | 488 | 883 | 1199 |
| f7900dfc | *Fusarium solani* | 1627 | 417 | 354 | 144 | 113 | 599 |
| 39d3eb9a | *Fusarium sublunatum* | 3737 | 966 | 0 | 26 | 0 | 2745 |
| e533f2e1 | *Fusicolla acetilerea* | 379 | 0 | 54 | 104 | 199 | 22 |
| 9da795cb | *Gaertneriomyces semiglobifer* | 156 | 156 | 0 | 0 | 0 | 0 |
| ce3a80da | *Gilmaniella humicola* | 0 | 0 | 0 | 0 | 0 | 0 |
| 466cb503 | *Hannaella luteola* | 453 | 201 | 0 | 205 | 29 | 18 |
| 05c83732 | *Hannaella oryzae* | 1842 | 68 | 55 | 1374 | 0 | 345 |
| af89c123 | *Hannaella siamensis* | 267 | 90 | 75 | 60 | 24 | 18 |
| 42d11ac5 | *Heterophoma adonidis* | 903 | 0 | 13 | 0 | 890 | 0 |
| e38451b4 | *Iodophanus carneus* | 445 | 6 | 0 | 439 | 0 | 0 |
| 31752de2 | *Laetisaria arvalis* | 0 | 0 | 0 | 0 | 0 | 0 |
| 7efe2520 | *Lasiodiplodia theobromae* | 21 | 0 | 0 | 13 | 0 | 8 |
| eacf77f8 | *Latorua caligans* | 493 | 28 | 69 | 114 | 18 | 264 |
| 4051c6d3 | *Lectera longa* | 92 | 0 | 0 | 0 | 0 | 92 |
| b651b611 | *Leptodiscella* | 173 | 0 | 0 | 173 | 0 | 0 |
| 66d0f502 | *Leptodiscella africana* | 40 | 0 | 12 | 0 | 28 | 0 |
| d1b126ec | *Leptosphaerulina australis* | 39930 | 758 | 4553 | 2376 | 29557 | 2686 |
| 0a92cbef | *Leptospora thailandica* | 34 | 0 | 34 | 0 | 0 | 0 |
| d484c145 | *Lipomyces kononenkoae* | 0 | 0 | 0 | 0 | 0 | 0 |
| 63d6b0a5 | *Mariannaea elegans* | 0 | 0 | 0 | 0 | 0 | 0 |
| 0ea20db6 | *Metacordyceps chlamydosporia* | 0 | 0 | 0 | 0 | 0 | 0 |
| 014027b4 | *Meyerozyma caribbica* | 13 | 0 | 0 | 13 | 0 | 0 |
| a4b8636f | *Minimedusa polyspora* | 15 | 0 | 0 | 0 | 15 | 0 |
| d2a11c63 | *Mortierella ambigua* | 0 | 0 | 0 | 0 | 0 | 0 |
| a4f3c1b6 | *Mortierella macrocystis* | 93 | 0 | 0 | 0 | 36 | 57 |
| 9de5f7a1 | *Mortierella wolfii* | 29 | 0 | 0 | 29 | 0 | 0 |
| 880f1ac6 | *Myriococcum thermophilum* | 0 | 0 | 0 | 0 | 0 | 0 |
| 0d04c87a | *Myrothecium gramineum* | 80 | 0 | 0 | 58 | 15 | 7 |
| 51182971 | *Myrothecium inundatum* | 649 | 140 | 42 | 247 | 37 | 183 |
| 28a26da1 | *Myrothecium verrucaria* | 7086 | 115 | 442 | 1463 | 787 | 4279 |
| 6f7e1743 | *Naganishia albida* | 108 | 0 | 108 | 0 | 0 | 0 |
| ad8dc8d2 | *Naganishia diffluens* | 7878 | 311 | 164 | 1145 | 5972 | 286 |
| a707102a | *Neurospora terricola* | 244 | 26 | 0 | 141 | 77 | 0 |
| aa7aa229 | *Nigrospora oryzae* | 143 | 111 | 0 | 32 | 0 | 0 |
| 9e6a0d85 | *Ochroconis minima* | 90 | 0 | 0 | 80 | 10 | 0 |
| e4b70f20 | *Papiliotrema flavescens* | 101 | 10 | 42 | 0 | 49 | 0 |
| 987bafe6 | *Papiliotrema laurentii* | 471 | 23 | 333 | 58 | 57 | 0 |
| e5621848 | *Papiliotrema rajasthanensis* | 31 | 0 | 0 | 0 | 0 | 31 |
| 4741fb04 | *Papiliotrema terrestris* | 17 | 0 | 0 | 6 | 11 | 0 |
| 3cfc6301 | *Paraconiothyrium* | 87 | 0 | 26 | 37 | 24 | 0 |
| 89d2172e | *Paracremonium inflatum* | 72 | 0 | 0 | 0 | 68 | 4 |
| 3b82b62d | *Penicillium montanense* | 0 | 0 | 0 | 0 | 0 | 0 |
| 10bcad9a | *Periconia byssoides* | 264 | 7 | 76 | 100 | 11 | 70 |
| 06ee7721 | *Peziza ripensis* | 0 | 0 | 0 | 0 | 0 | 0 |
| 04983f52 | *Phaeosphaeria podocarpi* | 1011 | 15 | 0 | 438 | 558 | 0 |
| 13a164f7 | *Phialocephala humicola* | 0 | 0 | 0 | 0 | 0 | 0 |
| 81e9b1b3 | *Phoma herbarum* | 35834 | 15498 | 48 | 16756 | 398 | 3134 |
| 9719d79a | *Phomatospora biseriata* | 0 | 0 | 0 | 0 | 0 | 0 |
| e1f3d0e7 | *Plectosphaerella cucumerina* | 203 | 30 | 0 | 81 | 27 | 65 |
| 29f7b5b8 | *Plectosphaerella niemeijerarum* | 634 | 0 | 0 | 528 | 46 | 60 |
| db5cfc3e | *Poaceascoma helicoides* | 53 | 0 | 0 | 0 | 53 | 0 |
| f5a455a2 | *Podospora communis* | 95 | 0 | 84 | 0 | 0 | 11 |
| 294f5b65 | *Podospora dimorpha* | 164 | 0 | 0 | 160 | 0 | 4 |
| 77b5f6a5 | *Preussia persica* | 4429 | 0 | 0 | 3911 | 490 | 28 |
| 94b8890c | *Preussia terricola* | 54 | 54 | 0 | 0 | 0 | 0 |
| 04454bd9 | *Pseudallescheria boydii* | 17 | 17 | 0 | 0 | 0 | 0 |
| a678a508 | *Pseudopithomyces chartarum* | 7273 | 753 | 454 | 844 | 243 | 4979 |
| 0c60ce96 | *Pyrenochaetopsis leptospora* | 138 | 0 | 0 | 35 | 103 | 0 |
| 79e8b586 | *Remotididymella anthropophila* | 75540 | 1043 | 3645 | 42011 | 8958 | 19883 |
| 605f7c29 | *Rhizophlyctis rosea* | 1308 | 0 | 722 | 30 | 556 | 0 |
| 9561ac02 | *Rhodotorula diobovata* | 266 | 0 | 0 | 45 | 221 | 0 |
| 37afa533 | *Rhodotorula kratochvilovae* | 18 | 18 | 0 | 0 | 0 | 0 |
| b1a8382d | *Rhodotorula mucilaginosa* | 5562 | 195 | 24 | 164 | 5115 | 64 |
| daa45bee | *Rhodotorula toruloides* | 129 | 0 | 36 | 0 | 0 | 93 |
| 3342c41c | *Robillarda sessilis* | 46 | 0 | 40 | 0 | 6 | 0 |
| 1f72fda7 | *Roussoella solani* | 159 | 18 | 4 | 137 | 0 | 0 |
| d3a72aba | *Russula cyanoxantha* | 0 | 0 | 0 | 0 | 0 | 0 |
| f82e704a | *Russula nigricans* | 1400 | 1400 | 0 | 0 | 0 | 0 |
| b27771fc | *Saitozyma flava* | 238 | 0 | 0 | 107 | 131 | 0 |
| 07d31ee6 | *Saitozyma podzolica* | 193 | 62 | 70 | 0 | 0 | 61 |
| 0ce2d481 | *Sakaguchia oryzae* | 330 | 0 | 0 | 0 | 330 | 0 |
| 876464fe | *Scolecobasidium humicola* | 100 | 0 | 78 | 0 | 22 | 0 |
| 9246cf4e | *Setophaeosphaeria badalingensis* | 0 | 0 | 0 | 0 | 0 | 0 |
| a8cb7e74 | *Setophoma vernoniae* | 613 | 517 | 25 | 0 | 0 | 71 |
| a887a4d9 | *Spizellomyces acuminatus* | 118 | 118 | 0 | 0 | 0 | 0 |
| 3cb5e08c | *Spizellomyces palustris* | 4463 | 2559 | 0 | 140 | 1764 | 0 |
| 2243b00a | *Stachybotrys chlorohalonata* | 0 | 0 | 0 | 0 | 0 | 0 |
| 78065ef9 | *Stachybotrys limonispora* | 15 | 7 | 0 | 0 | 0 | 8 |
| 24b55dff | *Stachybotrys microspora* | 37 | 0 | 6 | 31 | 0 | 0 |
| 3f0e0b41 | *Stagonosporopsis cucurbitacearum* | 3461 | 61 | 430 | 950 | 913 | 1107 |
| 91dc45a8 | *Stephanonectria keithii* | 389 | 0 | 0 | 389 | 0 | 0 |
| 1ceffd8d | *Strelitziana africana* | 653 | 0 | 122 | 0 | 531 | 0 |
| 4c19d908 | *Subulicystidium perlongisporum* | 0 | 0 | 0 | 0 | 0 | 0 |
| ea1b01e9 | *Symmetrospora symmetrica* | 188 | 0 | 3 | 185 | 0 | 0 |
| febcb3ed | *Talaromyces stollii* | 0 | 0 | 0 | 0 | 0 | 0 |
| 4dfde891 | *Tetragoniomyces uliginosus* | 20 | 0 | 20 | 0 | 0 | 0 |
| 397010ec | *Thanatephorus cucumeris* | 3267 | 0 | 0 | 403 | 1047 | 1817 |
| 41fd260d | *Trichoderma asperellum* | 36 | 0 | 0 | 36 | 0 | 0 |
| bdc77c47 | *Trichoderma brevicompactum* | 5 | 0 | 0 | 0 | 0 | 5 |
| 37aa5ee8 | *Trichoderma harzianum* | 3 | 0 | 3 | 0 | 0 | 0 |
| b14292d7 | *Trichoderma longibrachiatum* | 57 | 55 | 0 | 0 | 0 | 2 |
| eb641049 | *Trichosporon asahii* | 87 | 0 | 87 | 0 | 0 | 0 |
| 977d5d91 | *Triparticalcar equi* | 0 | 0 | 0 | 0 | 0 | 0 |
| 95d0adcc | *Ustilago cynodontis* | 73 | 0 | 0 | 73 | 0 | 0 |
| f403a941 | *Verticillium dahliae* | 0 | 0 | 0 | 0 | 0 | 0 |
| df839402 | *Westerdykella centenaria* | 1167 | 24 | 0 | 0 | 0 | 1143 |
| 608d8216 | *Westerdykella cylindrica* | 535 | 0 | 444 | 13 | 45 | 33 |
| 33a845c4 | *Wojnowiciella dactylidis* | 607 | 8 | 45 | 548 | 0 | 6 |
| 44a84e2b | *Zopfiella pilifera* | 73 | 0 | 73 | 0 | 0 | 0 |

**Supplementary References:**

1. Oksanen, F. J. et al. Vegan: Community Ecology Package. R package Version 2.4-3 (2017) https://CRAN.R-project.org/package=vegan.
2. Revankar, S. G. & Sutton. D. A. Melanized fungi in human disease. *Clin. Microbiol. Rev.* **23,** 884-928 (2010).
3. Bessadat, N. et al. Occurrence of Leaf Spot Disease Caused by *Alternaria crassa* (Sacc.) Rands on Jimson Weed and Potential Additional Host Plants in Algeria. *Plant Pathol J.* **36,** 179 (2020).
4. Velázquez-Jiménez, Y., Hernández-Castro, R., Romero-Romero, L., Salas-Garrido, C. G., & Martínez-Chavarría, L. C. Feline Phaeohyphomycotic Cerebellitis Caused by *Cladosporium cladosporioides*-complex: Case Report and Review of Literature. *J. Comp. Pathol.* **170,** 78-85 (2019).
5. Garcia-Hermoso, D. et al. Diversity of coelomycetous fungi in human infections: a 10-y experience of two European reference centres. *Fungal Biol.* **123,** 341-349 (2019).
6. Salah, H. et al. Phylogenetic diversity of human pathogenic *Fusarium* and emergence of uncommon virulent species. *J. Infect.* **71,** 658-666 (2015).
7. van Diepeningen. A. D. et al. Spectrum of *Fusarium* infections in tropical dermatology evidenced by multilocus sequencing typing diagnostics. *Mycoses* **58,** 48-57 (2015).
8. Valenzuela-Lopez, N. et al. Coelomycetous fungi in the clinical setting: morphological convergence and cryptic diversity. *J. Clin. Microbiol.* **55,** 552-567 (2017).
9. Rameshkumar, G., Sikha, M., Ponlakshmi, M., & Lalitha, P. A rare case of *Myrothecium* species causing mycotic keratitis: diagnosis and management. *Med. Mycol. Case Rep.* **25,** 53-55 (2019).
10. Khawcharoenporn, T., Apisarnthanarak, A., & Mundy, L. M. Non-neoformans cryptococcal infections: a systematic review. *Infection* **35,** 51 (2007).
11. Hurst, C. J. Dirt and disease: the ecology of soil fungi and plant fungi that are infectious for vertebrates. In *Understanding terrestrial microbial communities* 289-405 Springer, Cham (2019).
12. Choe, Y. J. et al. *Cryptococcus albidus* Fungemia in an immunosuppressed child: case report and systematic literature review. *J. Pediatr. Infect. Dis.* **9,** 100-105 (2020).
13. Bandalizadeh. Z. et al. *Cryptococcus* and cryptococcosis in Iran during 1969–2019: A systematic review and meta-analysis. *J. Mycol. Med.* **30,** 100917 (2020).
14. Valenzuela-Lopez. N. et al. Coelomycetous Dothideomycetes with emphasis on the families Cucurbitariaceae and Didymellaceae. *Stud. Mycol.* **90,** 1-69 (2018).
15. Wirth, F. & Goldani, L. Z. Epidemiology of *Rhodotorula*: an emerging pathogen. *Interdiscip. Perspect. Infect. Dis.* (2012).
16. Ioannou, P., Vamvoukaki, R., and Samonis, G. *Rhodotorula* species infections in humans: A systematic review. *Mycoses*, **62,** 90-100 (2019).
